# U5 snRNP Core Proteins Are Key Components of the Defense Response against Viral Infection through Their Roles in Programmed Cell Death and Interferon Induction

**DOI:** 10.3390/v14122710

**Published:** 2022-12-03

**Authors:** Simon Boudreault, Guy Lemay, Martin Bisaillon

**Affiliations:** 1 Département de Biochimie et de Génomique Fonctionnelle, Faculté de Médecine et des Sciences de la Santé, Université de Sherbrooke, Sherbrooke, QC J1E 4K8, Canada; 2 Département de Microbiologie, Infectiologie et Immunologie, Faculté de Médecine, Université de Montréal, Montréal, QC H3C 3J7, Canada

**Keywords:** reovirus, spliceosome, U5 snRNP, EFTUD2, PRPF8, SNRNP200, apoptosis, necroptosis, interferon response, viral replication

## Abstract

The spliceosome is a massive ribonucleoprotein structure composed of five small nuclear ribonucleoprotein (snRNP) complexes that catalyze the removal of introns from pre-mature RNA during constitutive and alternative splicing. EFTUD2, PRPF8, and SNRNP200 are core components of the U5 snRNP, which is crucial for spliceosome function as it coordinates and performs the last steps of the splicing reaction. Several studies have demonstrated U5 snRNP proteins as targeted during viral infection, with a limited understanding of their involvement in virus–host interactions. In the present study, we deciphered the respective impact of EFTUD2, PRPF8, and SNRNP200 on viral replication using mammalian reovirus as a model. Using a combination of RNA silencing, real-time cell analysis, cell death and viral replication assays, we discovered distinct and partially overlapping novel roles for EFTUD2, PRPF8, and SNRNP200 in cell survival, apoptosis, necroptosis, and the induction of the interferon response pathway. For instance, we demonstrated that EFTUD2 and SNRNP200 are required for both apoptosis and necroptosis, whereas EFTUD2 and PRPF8 are required for optimal interferon response against viral infection. Moreover, we demonstrated that EFTUD2 restricts viral replication, both in a single cycle and multiple cycles of viral replication. Altogether, these results establish U5 snRNP core components as key elements of the cellular antiviral response.

## 1. Introduction

During viral infection, the interferon (IFN) pathway is the principal cellular response to fight back viruses and signal an infection to the immune system [1]. Pathogen-associated molecular patterns (PAMP), such as double-stranded RNA (dsRNA) and 5′-triphosphate single-stranded RNA, are detected by pattern-recognition receptors (e.g. RIG-I, MDA5, TLR3, etc.) and through a complex signaling cascade culminate with the production of IFN. Upon secretion, IFN can act in a paracrine fashion on uninfected cells to prepare them prior to getting infected, or in an autocrine fashion on infected cells to stimulate them in their fight against viral infection. The interaction of IFN with its receptor triggers the expression of hundreds of interferon-stimulated genes (ISG), which are the effectors of the cellular antiviral response [1,2]. On the other end of the spectrum, programmed cell death is another means for the cell to hamper viral replication by killing the cell before the virus can replicate efficiently. Diverse programs of cell death allow cells to perform this altruistic death in favor of overall survival of the greater number [3]. The most well-known is apoptosis, characterized by caspase activation and shrinkage of the cell [3]. Apoptosis is notably recognized as a “clean” death, as there is no leakage of the cellular content, and the remaining apoptotic bodies are swallowed by macrophages with limited inflammation. Conversely, necroptosis is emerging as a key programmed cell death to trigger inflammation, as it results in leakage of the cellular content, which is highly immunogenic [3]. The sensors of necroptosis (i.e., RIPK1, ZBP1 (formerly DAI), and TRL3/TRL4) all activate RIPK3, which is the primary regulator of this cell death program. Upon phosphorylation, RIPK3 is activated and phosphorylates MLKL, the main effector of necroptosis. MLKL phosphorylation leads to its trimerization and the formation of pores in the cellular membrane, which results in the leakage of the cytoplasmic content into the extracellular space.

Recently, several viruses were shown to interact with the splicing machinery components and interfere with the splicing process during infection, with a limited understanding of the role of these spliceosomal proteins and splicing factors in virus–host interactions [4,5,6,7]. The spliceosome is a large ribonucleoprotein (RNP) structure composed of five small nuclear ribonucleoproteins complexes (snRNP; U1, U2, U4, U5, and U6), each formed by one small nuclear RNA and numerous proteins components. The spliceosome catalyzes the removal of introns for mRNA processing through constitutive splicing by recognizing key sequences in introns, such as the branch point and the polypyrimidine tract, and in exons, such as the 5′ splice site (5′-SS) and the 3′ splice site (3′-SS). The snRNP are sequentially recruited to the intron, starting with U1 binding the 5′-SS and U2 binding the branch point. Then, the U4/U6.U5 tri-snRNP is recruited to the assembling spliceosome, forming the precatalytic spliceosome, or complex B. U5 proteins EFTUD2 and SNRNP200 are critical to drive the reorganization of the complex, the release of U1 and U4 snRNP, and activation to excise the intron (B^act^) [8,9,10]. Two nucleophilic attacks, one from the branch site to the 5′-SS, and then from the 5′-SS to the 3′-SS, allow the removal of the intron. During these attacks, U5′s central protein PRPF8′s role is crucial, as it binds both the 5′-SS and 3′-SS [11]. Alternative splicing (AS), as opposed to constitutive splicing, arises from spliceosome assembly being stabilized or destabilized near weak splice sites by splicing factors bound to the pre-mRNA. These inhibitory and stimulatory signals allow the removal of exons, parts of exon, and even introns to be retained in the mature RNA. AS thus modifies the coding potential of the mRNA, and results in the formation of a mixed population of mature mRNAs arising from the same gene [12,13,14,15]. Emerging evidence seems to implicate U5 in cellular AS, as opposed to the previous vision that the B^act^ complex is committed to removing the intron and that U5 can thus only control constitutive splicing [5,16].

In the past few years, numerous viruses have been shown to modulate cellular AS upon infection [7,17,18,19]. The increasing importance of the impact of viruses on cellular AS has prompted us to study the impact of mammalian reovirus (MRV) infection on cellular AS. MRV is a dsRNA virus from the *Reoviridae* family which has been fundamental to our comprehension of the basis of virus replication, such as internalization, uncoating, transcription, RNA cap synthesis, translation, and virus–host interactions [20,21,22,23]. The genome of MRV is composed of ten dsRNA segments encoding eight structural proteins and four non-structural proteins and is protected by a double-layered capsid. Previously, we and others have demonstrated that MRV infection induces drastic changes in cellular AS, identifying more than 200 cellular AS events impacted by MRV infection [23,24]. We recently showed that MRV’s μ2 protein is the primary viral protein involved in this modulation [5]. μ2 impacts cellular AS by interacting with U5 snRNP proteins EFTUD2, PRPF8, and SNRNP200, and reducing their levels during infection [5]. However, how are these U5 snRNP proteins involved in the fundamental aspect of viral replication, and what is the benefit for the virus to reduce these spliceosomal components remain unknown.

In the present study, we deciphered the impact of EFTUD2, PRPF8, and SNRNP200 silencing on viral replication using MRV as a model and discovered novel roles for EFTUD2 and SNRNP200 in cell survival, apoptosis, and necroptosis during viral infection. Moreover, EFTUD2 and PRPF8 control the induction of the IFN response pathway, establishing the U5 snRNP as a potent target for viruses to abrogate the cellular response mounted against infection. Finally, we demonstrated that EFTUD2 restricts the replication of MRV, both in a single cycle and multiple cycles of viral replication. Overall, these results establish U5 snRNP core components as key elements of the cellular antiviral response.

## 2. Materials and Methods

### 2.1. Cells, Viruses, and Treatments

Mouse L929 fibroblasts were originally obtained from the American Type Culture Collection (ATCC) and were grown in Eagle’s minimal essential medium (EMEM, Wisent) containing 5% fetal bovine serum (Wisent) and supplemented with 1% glutamine. MRV serotype 3 strain Dearing (T3/Human/Ohio/Dearing/55) was also originally obtained from ATCC and was propagated and titrated by TCID_50_ on L929 fibroblasts [25]. The laboratory stock of MRV type 3 (T3D^S^) was previously described [26,27], and rescued by reverse genetics following the introduction of the appropriate mutations in the plasmids encoding the virus from the original reverse genetics system [28]. The pan-caspase inhibitor zVAD-fmk (AdooQ Bioscience), the RIPK3 inhibitor GSK872 (Abcam) and the RIPK1 inhibitor GSK963 (Sigma) were resuspended at 10 mM in DMSO and used at various final concentrations. To elicit RIPK1-mediated necroptosis in L929 cells, cells were pretreated with zVAD-fmk at 100 μM for 1 h and then recombinant murine TNF-α (Peprotech) was directly added to the medium at 25 ng/mL. Recombinant mouse interferon-β (PBL Assay Science, #12401-1) was diluted in complete medium to the indicated concentration and cells were treated for 5 h.

### 2.2. Viral Infection

L929 cells were plated at a density of 7 × 10^4^ cells per square centimeter the day before being infected at a multiplicity of infection (MOI) of 0.1 or 3 TCID_50_ units per cell using standard procedures [25] where the inoculum is not removed after adsorption. Control L929 cells were seeded at the same density and mock-infected. Whenever cells needed to be transfected using siRNA first, the number of cells at the time of infection was evaluated based on the number of cells seeded for transfection, the time since transfection, and apparent confluency of the cells.

### 2.3. siRNA Transfection

L929 cells were plated in a 12-well plate at 100,000 cells/well and transfected on the following morning using 50 pmol of siRNA and 3.75 μL of RNAiMAX (ThermoFisher Scientific, Waltham, MA, USA), as per the manufacturer’s protocol. Ambion Silencer^®^ Select (catalog number 4390771) siRNA were used against EFTUD2 (ID: s74089); PRPF8 (ID: s101224); SNRNP200 (ID: s115821); and RIG-I (ID: s106375). The Silencer™ Select Negative Control No. 1 siRNA (#4390843) was used a negative control. Cells were incubated for 72 h before harvesting RNA or proteins for downstream analyses. For double knock-down (DKD), twice the quantity of siRNA was transfected to perform DKD alongside twice the volume of RNAiMAX; in the case of a single KD, siCTRL was added to match the total siRNA quantity of the DKD.

### 2.4. Real-Time Cell Analysis (RTCA)

The xCELLigence system (ACEA Biosciences) was used to monitor cell adherence and morphology in real time. E-plates were filled with 50 μL of complete culture medium per well, and the plates incubated for several hours at 37 °C with 5% CO_2_ to allow equilibration. Then, L929 cells were seeded at 5000 cells/well in 50 μL of complete medium and allowed to adhere overnight. The next morning, siRNA were transfected and the monitoring was resumed; 24 h later, the medium containing the siRNA complexes was removed and kept and cells were infected at a MOI of 3. Following adsorption, the medium containing the siRNA complexes was added back to the wells and plates were allowed to equilibrate approximately 15 min at 37 °C with 5% CO_2_ before the monitoring was resumed. The relative cell index was normalized to the 0 h time point, right after infection. Measurements were taken every 10 min for up to 100 h after infection. For the zVAD-fmk/TNFα treatment, cells were incubated for 72 h after siRNA transfection to allow an efficient depletion of the targeted proteins. Then, cells were pretreated for 1 h with 100 μM of zVAD-fmk, before medium was changed for zVAD-fmk (100 μM) and TNFα (25 ng/mL). A siCTRL condition was treated with only zVAD-fmk to confirm no cell death was induced in the absence of TNFα. Plates were allowed to equilibrate approximately 20 min at 37 °C with 5% CO_2_ before the monitoring was resumed. The relative cell index was normalized to the 0 h time point, right after TNFα was added. Measurements were taken every 15 min.

### 2.5. FACS Analysis

The cell culture medium was transferred to a new tube, and cells were harvested by trypsinization before being pooled with the culture medium. Upon centrifugation, the cell pellet was resuspended in 100 μL of annexin V binding buffer (10 mM HEPES, 140 mM NaCl, 2 mM CaCl_2_, pH 7.4) containing Hoechst (Invitrogen, 0.8 mg/mL) and propidium iodide (Invitrogen, 1 μg/mL). The cells were stained for 30 min at 37 °C with 5% CO_2_ before 400 μL of annexin V binding buffer was added and live cells were quantitated by FACS on a BD LSRFortessa. Cells were gated first using FSC/SSC to exclude cell debris, and then using Hoechst and SSC to only retain single cells. 10,000 events were acquired satisfying these criteria. Then, cell viability was quantitated by identifying live cells as both Hoechst positive and propidium iodide negative.

### 2.6. Methylene Blue Staining

L929 cells were seeded at 20,000 cells/well and transfected the following day with the appropriate siRNA. 24 h later, 1:1 dilutions of MRV were prepared, starting from a MOI of 12.5 to ≈0.01 (11 dilutions). A control well was left uninfected (mock). The transfection medium was removed from the plate and kept at 37 °C with 5% CO_2_; dilutions of the virus were added to the plates and followed by an incubation of 1 h at 4 °C. Transfection medium was then added back to their original wells, and plates were incubated for 48 h, 72 h, or 96 h at 37 °C with 5% CO_2_. Plates were fixed in 4% formaldehyde in PBS for 1 h, washed in PBS, and then stained with methylene blue (1% w/v methylene blue, 150 mM NaCl, 90% MeOH in water) for 1 h, which stains all adherent cells in the wells. Plates were washed once with PBS and twelve times with tap water and allowed to dry in a chemical hood. When dried, plates were imaged using a Quantum ST5 gel doc (Montreal Biotech, Vilber Lourmat, Dorval, QC, Canada). To allow for a precise quantification, cell bound methylene blue was resolubilized in 100 μL of 0.1 N HCl overnight and read at 665 nm in a 96-well plate reader. Normalization was realized by first setting the mock well in the siCTRL condition at 100% for each replicate; then, the mock for each other siRNA conditions was also normalized at 100%, to allow for correction of growth and confluency effects.

### 2.7. High-Throughput Multiplex Microscopy-Based Apoptosis Assay

The multiplex cell death phenotypic assay was performed as previously described [29,30]. L929 cells were seeded at 10,000 cells/well in black 96-well plates and transfected the next morning with the appropriate siRNA. Thirty hours later, medium was removed, kept at 37 °C with 5% CO_2_ and cells were mock-infected or infected with MRV at a MOI of 3. The initial medium was put back on the cells and infection was allowed to pursue for 40 h before the assay. The medium was removed and 50 μL of dye mix (Hoechst, Invitrogen, 0.8 mg/mL; calcein AM, Invitrogen, 0.5 μM; annexin V conjugated with Alexa Fluor 647, Invitrogen A23204, 2.85 μL/mL; propidium iodide, Invitrogen, 1 μg/mL; in annexin V binding buffer) was added to each well, and cells were stained 30 min at 37 °C, 5% CO_2_. The dye mix was removed and 50 µL of new annexin V binding buffer was added prior to imaging using the Operetta CLS High Content Analysis System (Perkin Elmer Life Sciences, Waltham, MA, USA). Nine different fields per well were imaged and imported into the Columbus software (PerkinElmer) for analysis. Each experiment included six biological replicates.

### 2.8. Caspase Activity Assay

Cas3/7 activity was measured using the Caspase-Glo^®^ 3/7 Assay System (Promega). Briefly, cells were treated with the siRNA for 55 h before being infected or mock-infected at a MOI of 3 for 40 h. Then, plates were allowed to equilibrate at room temperature for 10 min, and 100 μL of Caspase-Glo reagent was added to each well. Plates were shaken for 30 s, and read 30 min later in a TECAN SPARK multimode microplate reader for 5 s. First, mean background was subtracted to each measurement. Then, the first replicate of the siCTRL in the mock condition was fixed at 0%, and the first replicate of the siCTRL in the infected condition was fixed at 100%.

### 2.9. RNA Extraction

Total RNA samples were extracted with Qiazol^®^ as recommended by the manufacturer (Qiagen).

### 2.10. Reverse Transcription

Reverse transcription was performed on 2.2 µg total RNA with Transcriptor reverse transcriptase, random hexamers, dNTPs (Roche Diagnostics), and 10 units of RNAse OUT (Invitrogen) following the manufacturer’s protocol in a total volume of 20 µL.

### 2.11. qPCR

All forward and reverse primers were individually resuspended to 20–100 μM stock solution in Tris-EDTA buffer (IDT) and diluted as a primer pair to 1 μM in RNase DNase-free water (IDT). The complete list of qPCR primers used in this study is available in Supplementary Table S1. Quantitative PCR (qPCR) reactions were performed in 10 µL in 96-well plates on a CFX-96 thermocycler (BioRad) with 5 μL of 2 × iTaq Universal SYBR Green Supermix (BioRad), 10 ng (3 µL) cDNA, and 200 nM final (2 µL) primer pair solutions. The following cycling conditions were used: 3 min at 95 °C; 50 cycles: 15 s at 95 °C, 30 s at 60 °C, 30 s at 72 °C. Relative expression levels were calculated using the qBASE framework using *PSMC4*, *PUM1*, and *TXNL4B* as housekeeping genes. For all PCR run, control reactions performed in the absence of template were performed for each primer pair, and these were consistently negative. All qPCR data were generated following the MIQE guidelines [31].

### 2.12. Alternative Splicing PCR (AS-PCR)

PCR primer sequences were designed at the Université de Sherbrooke’s RNomics Platform using a custom software designed to optimize standard primer design criteria, and to certify target specificity using embedded NCBI Blast software. The primers were placed on exons flanking the alternative region to amplify both isoforms in the same PCR reaction. The complete list of AS-PCR primers used in this study is available in Supplementary Table S2. Design maps for the AS events analyzed in this study are shown in Supplementary Figure S10. All forward and reverse primers were individually resuspended to 20–100 μM stock solution in Tris-EDTA buffer (IDT) and diluted as a primer pair to 1.2 μM in RNase DNase-free water (IDT). End-point PCR reactions were done on 10 ng cDNA in 10 μL final volume containing 0.2 mM each dNTP, 1.5 mM MgCl_2_, 0.6 μM each primer, and 0.2 units of Platinum Taq DNA polymerase (Invitrogen). An initial incubation of 2 min at 95 °C was followed by 35 cycles at 94 °C 30 s, 55 °C 30 s, and 72 °C 60 s. The amplification was completed by a 2 min incubation at 72 °C. PCR reactions were carried on thermocyclers GeneAmp PCR System 9700 (ABI), and the amplified products were analyzed by automated chip-based microcapillary electrophoresis on LabChip GX Touch HT Nucleic Acid Analyzer (PerkinElmer). Amplicon sizing and relative quantitation were performed by the manufacturer’s software, before being uploaded to the LIMS database. The percent spliced-in (PSI) metric was used to quantitate the level of inclusion in these alternative splicing events. It represents the percent of the long form over total abundance for both the long and short forms. The formula is as follows:

$$PSI=\frac{Long\;form}{Long\;form+Short\;form}$$

### 2.13. Immunoblotting Analysis

The linearity of antibodies used in this study was first experimentally determined to allow the adequate quantification in the linear range of both the samples analyzed and the antibody used. Cells were scraped into the culture medium using the blunt end of a P1000 tip and pelleted at 3000 RPM for 10 min. Medium was removed and cells were lysed in RIPA Buffer (1% Triton X-100, 1% sodium deoxycholate, 0.1% SDS, 1 mM EDTA, 50 mM Tris-HCl pH 7.5 and complete protease inhibitor (ROCHE)) supplemented with 1X Halt™ Protease and Phosphatase Inhibitor Cocktail (ThermoFisher Scientific, Waltham, MA, USA) right before use. Sonication at 13% amplitude for 5 s, twice on ice was used to complete cell lysis and enhance the solubility of the samples. Debris were then pelleted at 13,000 RPM, 4 °C, 10 min. Lysates were dosed for total protein in triplicate using standard Bradford assay (ThermoFisher Scientific Pierce™ Coomassie (Bradford) Protein Assay Kit, Waltham, MA, USA). The right amount of protein was diluted with water and Laemmli 4− buffer (200 mM Tris-HCl pH 6.8, 40% glycerol, 1.47 M β-mercaptoethanol, 4% SDS, bromophenol blue) and heated 5 min at 95 °C. Samples were loaded on 10% SDS-polyacrylamide gels alongside BLUelf protein ladder (FroggaBio) and electrophoresis was carried out at 70 volts until samples entered the resolving gel. Electrophoresis was allowed to proceed at 100 volts until complete migration. Proteins were transferred onto a polyvinylidene difluoride (PVDF) membrane at 4 °C, 75 min, 100 volts. Membranes were blocked in 5% non-fat milk in TBS-T (10 mM Tris-HCl pH 8.0, 220 mM NaCl, 0.1% Tween 20), 1 h at room temperature. The commercial antibodies used in this study are the following: Actin (Sigma, A5441, 1:10,000), MLKL phosphorylated on S345 (Cell Signaling Technology, #37333, 1:1000), MLKL (Cell Signaling Technology, #37705, 1:1000), RIPK1 (Cell Signaling Technology, #37333) and RIPK3 (Cell Signaling Technology, #37333). The σ3 antibody is the supernatant from a mouse hybridoma cell line expressing the monoclonal antibody 4F2 and was diluted 1:100 [32]. Membranes were incubated overnight with the appropriate antibody in 2.5% milk/PBS, except for pMLKL and MLKL antibodies which were diluted with 5% bovine serum albumin in TBS-T. Membranes were washed three times in TBS-T and incubated with a horse anti mouse-HRP secondary antibody diluted 1:5000 (Cell Signaling Technologies, 7076) or goat anti rabbit-HRP secondary antibody diluted 1:10,000 (Abcam, ab205718) during 1 h 30 min at room temperature. Membranes were washed again three times with TBS-T and once with PBS. Bound antibodies were revealed using Clarity ECL Western blotting substrates (Bio-Rad Laboratories, Hercules, CA, USA) and scanned with an ImageQuant LAS4000 (GE Healthcare Life Science). For quantification, HRP was inactivated using 30% H_2_O_2_ for 30 min, washed twice in PBS, and membranes were blocked again and probed for actin. For pMLKL/MLKL WB, membranes were stripped after the actin to probe total MLKL. Membranes were incubated 15 min at 56 °C in stripping buffer (7.25 mM Tris-HCL pH 7.5, 0.1% SDS, 0.1% β-mercaptoethanol) before being blocked again and follow the usual procedure. All Western blots were performed three times, and a representative result is presented in the article. Uncropped Western blots are available in the Supplementary Figure S11.

### 2.14. Infectious Viral Titers Using TCID_50_

For siRNA depletion, L929 cells were seeded in 24-well plates at 100,000 cells/well (MOI of 0.1) or 40,000 cells/well (MOI of 3). On the following morning, cells were transfected with the appropriate siRNA. Infections were performed 24 h later (MOI of 0.1); the transfection medium was kept and put back on the cell immediately after viral adsorption, or 55 h later (MOI of 3) and the transfection medium discarded. For chemical treatments, L929 cells were seeded in 24-well plates at 125,000 cells/well the day before infection, and respective concentration of the drugs were added after infection the following day. Upon incubation for the appropriate time at 37 °C with 5% CO_2_, samples were harvested by three freezing-thawing cycle (−80 °C, 37 °C), aliquoted, and infectious viral titers were determined by TCID_50_ on L929 fibroblasts, as described before [25].

### 2.15. Data Analysis and Statistical Analyses

Image quantitation was done using the ImageJ software (Fiji). All statistical analyses were conducted with the GraphPad software. In some case, the variance ratio between samples was superior to 1.5, and thus Brown-Forsythe and Welch one-way ANOVA was applied [33]. All results presented in this article are mean ± standard deviation.

### 2.16. Data Availability

All data generated or analyzed during this study are included in this published article (and its Supplementary Information Files).

## 3. Results

### 3.1. EFTUD2 and SNRNP200 Are Required for Cell Death after Infection

**Figure 1:**
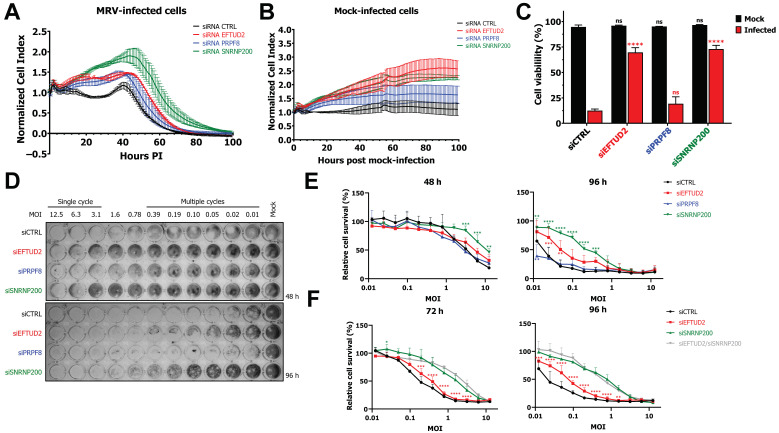
EFTUD2 and SNRNP200 KD protect cells from death after MRV infection. (**A**) RTCA of MRV-infected L929 cells (MOI of 3) transfected with a control siRNA or EFTUD2, PRPF8, or SNRNP200 specific siRNA. Measurements were made every 10 min. n = 3, biological replicates. (**B**) RTCA of mock-infected L929 cells transfected with a control siRNA or EFTUD2, PRPF8, or SNRNP200 specific siRNA. Measurements were made every 10 min. n = 3, biological replicates. (**C**) Cell viability in mock- or MRV-infected L929 cells. Cells were treated with the siRNA for 55 h before being mock-infected or infected at a MOI of 3 for 65 h. Cell viability was measured using FACS by staining with Hoechst and propidium iodide; live cells were selected as Hoechst positive and propidium iodide negative. n = 3, biological replicates, two-way ANOVA with Dunnett’s multiple comparisons test against the siCTRL treated cells for each condition (mock in black, infected in red; ns, *p* > 0.05; ****, *p* ≤ 0.0001). (**D**) Representative images of methylene blue staining of control or EFTUD2, PRPF8, or SNRNP200-depleted L929 cells infected with 1:1 dilutions of MRV at 48 h or 96 h post-infection. (**E**) Quantification of cell-bound methylene blue stain for three independent experiments. n = 3, biological replicates, two-way ANOVA with Dunnett’s multiple comparisons test against the control siRNA condition (**, *p* ≤ 0.01; ***, *p* ≤ 0.001; ****, *p* ≤ 0.0001); if not indicated, results are non-statistically significant. (**F**) Methylene blue staining of control, EFTUD2, SNRNP200, or EFTUD2/SNRNP200-depleted L929 cells infected with 1:1 dilutions of MRV at 72 h or 96 h post-infection. Twice the quantity of siRNA was transfected to perform DKD; in the case of single KD, siCTRL was added to match the total siRNA quantity of the DKD. The quantification of cell-bound methylene blue stain is shown for three independent experiments. n = 3, biological replicates, two-way ANOVA with Dunnett’s multiple comparisons test against the DKD condition, in gray (*, *p* ≤ 0.05; **, *p* ≤ 0.01; ***, *p* ≤ 0.001; ****, *p* ≤ 0.0001). If not indicated, results are non-statistically significant. The results of the comparison against the control siRNA are not indicated for clarity purposes.

As a first step towards understanding the role of U5 snRNP core proteins in viral replication, we used real-time cell analysis (RTCA) to profile the dynamics of MRV replication in siCTRL or EFTUD2, PRPF8, and SNRNP200 knocked-down (KD) cells. We previously demonstrated efficient KD of these proteins ranging from 50 to 70% using the same experimental conditions [5]. Nevertheless, we still validated using an orthogonal assay, namely qPCR, that the depletion was still both efficient and specific (Supplementary Figure S1). Murine L929 cells were used, as these are a well-known and established model for MRV infection, and our previous studies regarding the impact of MRV on cellular AS and the involvement of U5 snRNP proteins were done using these cells [5,23]. To perform RTCA, L929 cells were seeded and allowed to adhere overnight before transfecting them with the appropriate siRNA. After an incubation of 24 h with the siRNA, cells were mock-infected or infected with MRV at a multiplicity of infection (MOI) of 3, and the normalized cell index was followed throughout the infection. As seen in the control condition, the normalized cell index is stable up to 30 h, after which an increase is apparent before a constant decrease corresponding to the lysis of cells after infection is observed (Figure 1A). This curve is typical of infected cells [34,35,36,37,38]. However, when components of the U5 snRNP were depleted, the curves were shifted towards the right, suggesting that cells were surviving longer after infection. Moreover, the effect of SNRNP200 was still observable at a MOI of 50; however, the impact for EFTUD2 and PRPF8 at higher MOI was less clear (Supplementary Figure S2). We also validated that the depletion of EFTUD2, PRPF8, and SNRNP200 was not decreasing cell viability, and that cell survived and even expanded throughout the course of the experiment in the absence of infection (Figure 1B). Moreover, this increase in survival cannot be linked to a decrease in viral replication linked to the KD, as we previously demonstrated that both viral proteins and RNA are not decreased by EFTUD2, PRPF8, and SNRNP200 KD [5]. These results suggest that U5 snRNP proteins might control cell death and/or cell survival during MRV infection. To validate these results, we quantified cell viability using FACS by staining with Hoechst (nucleus) and propidium iodide (dead cells) (Figure 1C). This assay confirmed a significant increase in cell survival, as measured by the percentage of cells negative for propidium iodide compared to total cell count, upon KD of EFTUD2 and SNRNP200 and no effect of PRPF8 on cell survival during MRV infection.

To further validate the effect of EFTUD2 and SNRNP200 on cell survival, we exploited a previously described methylene blue staining assay using MRV 1:1 dilutions that allows for quantifying adherent cells in the wells [26,27,39,40]. L929 cells were treated with control, EFTUD2, PRPF8, or SNRNP200 siRNA and then infected with MRV dilutions, ranging from a MOI of approximately 0.01 to 12.5. This experimental workflow allows for profiling of both a single cycle of replication (MOI of 3 to 12.5) and multiple cycles (MOI < 0.1) by changing the incubation time. At 48 h, most cells treated with siCTRL or siPRPF8 were dead at the highest MOI; however, cells were surviving better when treated with the siRNA against SNRNP200 and EFTUD2 (Figure 1D). This protection from cell death was even more striking at 96 h when looking at multiple cycles of replication; cells knocked-down for SNRNP200 and EFTUD2 survived several dilutions higher than siCTRL or siPRPF8-treated cells (Figure 1D). To quantify this phenomenon, we measured cell-bound methylene blue using spectrophotometry in biological triplicates, both at 48 h and 96 h (Figure 1E). In average, 20–30% of cells survived at the highest MOI when treated with siCTRL, siEFTUD2, or siPRPF8 at 48 h. This survival increased to 45% in the case of the siSNRNP200. At 96 h, 90% (siSNRNP200) and 75% (siEFTUD2) of cells at the lowest MOI were still alive, compared to 35% with the siRNA against the control or PRPF8. Finally, we wondered if these effects of EFTUD2 and SNRNP200 were independent or merely reflecting the involvement of both proteins in the same cellular process. To ask this question, we KD EFTUD2, SNRNP200, and both proteins altogether and looked if the double knock-down (DKD) could confer synergetic protection from MRV-mediated cell death. Since twice the amount of siRNA was transfected to perform DKD, cells survived longer than in the previous assay at earlier timepoints; we thus performed the DKD at 72 h and 96 h. The DKD of EFTUD2 and SNRNP200 did not protect cells from death more than the simple KD of SNRNP200 at both 72 h and 96 h (Figure 1F). This experiment led us to conclude that the impact of EFTUD2 and SNRNP200 on cell survival are redundant and likely to involve a similar mechanism. Together, these data show that U5 snRNP proteins EFTUD2 and SNRNP200 are involved in cell survival and/or cell death after infection with MRV.

### 3.2. EFTUD2 and SNRNP200 Knock-Down Affects Apoptosis

**Figure 2:**
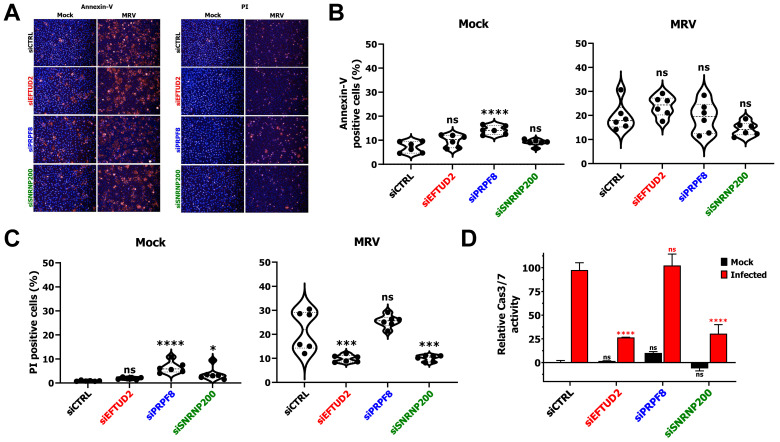
EFTUD2 and SNRNP200 depletion hamper apoptosis. (**A**) Representative set of images for the high-throughput multiplex microscopy-based apoptosis assay. On the left, Annexin-V signal is shown in red, representative of apoptotic cells. On the right, propidium iodide signal is shown in red, representative of dead cells. On both sides, the nuclei are stained with Hoechst and shown in blue. (**B**) Percent of mock- or MRV-infected L929 cells staining positive for Annexin-V upon depletion of EFTUD2, PRPF8, or SNRNP200. Cells were infected (MOI of 3) or mock-infected, and the percent of Annexin-V positive cells was calculated 40 h later. n = 6, biological replicates, one-way ANOVA with Dunnett’s multiple comparisons test against the siCTRL treated cells (ns, *p* > 0.05; ****, *p* ≤ 0.0001). (**C**) Percent of dead cells upon depletion of EFTUD2, PRPF8, or SNRNP200. Cells were infected (MOI of 3) or mock-infected, and the percent of propidium iodide positive cells was calculated at 40 h post infection. n = 6, biological replicates, one-way ANOVA with Dunnett’s multiple comparisons test against the siCTRL treated cells (ns, *p* > 0.05; *, *p* ≤ 0.05; ***, *p* ≤ 0.001; ****, *p* ≤ 0.0001). (**D**) Relative Cas3/7 activity for mock- and MRV-infected L929 cells. Cells were treated with the siRNA for 55 h before being infected or mock-infected at a MOI of 3 for 40 h. Cas3/7 activity was measured using the Caspase-Glo^®^ 3/7 Assay System. The first replicate of the siCTRL in the mock condition was fixed at 0%, and the first replicate of the siCTRL in the infected condition was fixed at 100%. n = 3, biological replicates, two-way ANOVA with Dunnett’s multiple comparisons test against the siCTRL treated cells for each condition (mock in black, infected in red; ns, *p* > 0.05; ****, *p* ≤ 0.0001).

After infection with MRV, both apoptosis [41,42,43] and necroptosis [44,45] can be induced and result in cell death and lysis. We first questioned if KD of EFTUD2 and SNRNP200 impacted apoptosis to enhance cell survival after MRV infection by using a high-throughput microscopy-based assay. L929 cells were treated with the appropriate siRNA and infected with MRV for 40 h, just before cells started to die under these conditions (see Figure 1A). Cells were then stained with Annexin-V conjugated to AlexaFluor-647 (early apoptosis), propidium iodide (dead cells), calcein AM (cytoplasm) and Hoechst (nucleus) in 96-well plates, as previously described [29,30]. Plates were imaged using a 96-well plate microscope, and positive cells were automatically counted. In mock condition, cells exhibited low levels of apoptosis, with only a modest but significant impact of the PRPF8 siRNA (Figure 2A,B). Moreover, when cells were infected with MRV, there was no significant reduction of apoptotic positive cells, as measured by this assay, with any of the siRNA transfected compared to the control siRNA (Figure 2A,B). This result suggests that apoptosis normally proceeds, even in EFTUD2- and SNRNP200-depleted cells. In addition, staining for dead cells using propidium iodide revealed a significant decrease in dead cells when EFTUD2 and SNRNP200 were KD, further strengthening our previous finding that these KD protect cells from MRV-mediated death (Figure 1C and Figure 2A,C). Once again, a small but significant impact could be observed in PRPF8-depleted mock cells and also in the SNRNP200-depleted condition (Figure 2C). To validate that apoptosis was indeed induced, we further quantified the activity of caspase 3 and 7, the effector caspases of apoptosis, using a luminescence-based assay. Surprisingly, this assay revealed a 75% reduction in Cas3/7 activity in MRV-infected cells when either EFTUD2 or SNRNP200 was depleted (Figure 2D). Since the extracellular exposure of phosphatidylserine is induced in other programmed cell death pathways than apoptosis [46,47], this latest result suggests that even though cells present extracellular phosphatidylserine (Figure 2B), the activity of effector caspases are actually reduced. Apoptosis is thus impaired upon EFTUD2 and SNRNP200 depletion in MRV-infected cells, and both the depletion of EFTUD2 and SNRNP200 likely protects cells from MRV’s induced apoptosis.

### 3.3. EFTUD2 and SNRNP200 Are Required for Necroptosis

**Figure 3:**
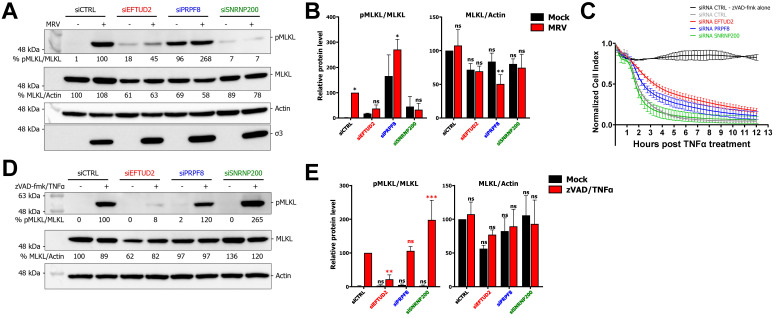
Depletion of EFTUD2 and SNRNP200 impacts necroptosis. (**A**) Western blot of phosphorylated MLKL (pMLKL) and total MLKL in mock- or MRV-infected L929 cells treated with control or EFTUD2, PRPF8, or SNRNP200 specific siRNA. Cells were infected at an MOI of 3 and incubated for 40 h before proteins were harvested. Actin was used as a loading control and the MRV σ3 viral protein was used to validate the infection. Relative quantitation is shown for pMLKL/MLKL and MLKL/Actin. (**B**) Quantification and statistical analysis of three independent Western blots. For pMLKL/MLKL, the infected cells were compared to their respective non-infected siRNA condition using a two-way ANOVA with Šídák’s multiple comparisons test; for MLKL/Actin, all conditions were compared to the siCTRL mock condition using a one-way ANOVA with Dunnett’s multiple comparisons test. n = 3, biological replicates (ns, *p* > 0.05; *, *p* ≤ 0.05; **, *p* ≤ 0.01). (**C**) RTCA of zVAD-fmk/TNFα treated L929 cells transfected with a control siRNA or EFTUD2, PRPF8, or SNRNP200 specific siRNA. A siCTRL condition was treated with only zVAD-fmk to confirm no cell death was induced in the absence of TNFα. Measurements were made every 15 min for 12 h. n = 3 for all conditions except for siRNA CTRL - zVAD-fmk alone (n = 2), biological replicates. (**D**) Western blot of phosphorylated MLKL (pMLKL) and total MLKL in control or EFTUD2, PRPF8, or SNRNP200-depleted L929 cells untreated or treated 4 h with zVAD/TNFα to induce necroptosis. Actin was used as a loading control. Relative quantitation is shown for pMLKL/MLKL and MLKL/Actin. (**E**) Quantification and statistical analysis of three independent Western blot. For pMLKL/MLKL, the zVAD/TNFα treated cells were compared to the zVAD/TNFα siCTRL condition (in red) and the same was done for the untreated cells (in black) using a two-way ANOVA with Šídák’s multiple comparisons test; for MLKL/Actin, all conditions were compared to the siCTRL mock condition using a one-way ANOVA with Dunnett’s multiple comparisons test. n = 3, biological replicates (ns, *p* > 0.05; **, *p* ≤ 0.01; ***, *p* ≤ 0.001).

Next, we turned to the potential impact of EFTUD2 and SNRNP200 on necroptosis, which is also induced in MRV-infected cells [44,45]. The striking effect of U5 snRNP proteins KD on cell survival during MRV infection suggests that these proteins of the U5 snRNP (i.e., EFTUD2 and SNRNP200) might affect more than simply apoptosis to confer such a drastic enhancement in cell survival. The effector of necroptosis, MLKL, is phosphorylated on S345 by RIPK3, which allows for its trimerization and the formation of pores that leak the cellular content into the extracellular space [48,49]. We thus quantitated the levels of phosphorylated MLKL (pMLKL) as an indicator of necroptosis in mock- and MRV-infected cells treated with control or EFTUD2, PRPF8 or SNRNP200 specific siRNA (Figure 3A). Upon EFTUD2 or SNRNP200 KD, there was a defect in the phosphorylation of MLKL in infected cells. Quantification of multiple Western blots (WB) confirmed this significant absence of pMLKL induction in EFTUD2 and SNRNP200-depleted cells after MRV infection (Figure 3B). Surprisingly, PRPF8 KD led to a considerable amount of pMLKL in the absence of infection, revealing that the enhanced cell death observed with this KD (Figure 2B) could be attributable, at least in part, to necroptosis (Figure 3A,B). These results place U5 snRNP core components as regulators of necroptosis. We also observed some variations in the total protein levels of MLKL (Figure 3A), although there was significantly less MLKL only in the PRPF8 KD after MRV infection (Figure 3B).

Considering MRV-induced necroptosis is, at least in part, dependent on RIPK1 [44,45], we tested the impact of U5 core components KD with another RIPK1-dependent necroptosis stimulus. Necroptosis can be elicited in L929 cells by first blocking apoptosis using the pan-caspase inhibitor zVAD-fmk, and then stimulating the tumor necrosis factor receptor (TNFR) using TNFα, which elicits RIPK1-dependent necroptosis [48,49]. We first monitored EFTUD2-, PRPF8-, and SNRNP200-depleted cells treated with zVAD-fmk/TNFα using RTCA and observed results consistent with increased survival when EFTUD2 and PRPF8 levels were reduced (Figure 3C). However, using this stimulus, the depletion of SNRNP200 did not seem to confer any protection from necroptotic cell death when monitoring using RTCA. We then monitored EFTUD2-, PRPF8-, and SNRNP200-depleted cells treated with zVAD-fmk/TNFα using microscopy and confirmed that EFTUD2 depletion protected cells against zVAD-fmk/TNFα-mediated cell death, whereas SNRNP200 KD had no protective effect using this stimulus and even seemed to sensitize cells (Supplementary Figure S3). Using live-cell microscopy, we could not observe any impact of PRPF8 on cell survival following zVAD-fmk/TNFα necroptotic stimulus. To validate these results, we quantified the levels of pMLKL upon zVAD-fmk/TNFα stimulation in cells treated with control or EFTUD2, PRPF8, or SNRNP200 siRNA. The results revealed EFTUD2 depletion hampered the phosphorylation of MLKL upon zVAD-fmk/TNFα treatment, and that SNRNP200 enhanced MLKL phosphorylation (Figure 3D). Quantification of multiple WB confirmed a significant decrease of phosphorylated MLKL in EFTUD2-depleted cells, a significant increase in SNRNP200-depleted cells, while no impact of the PRPF8 depletion could be observed (Figure 3E). These results underline the opposite effect of SNRNP200 depletion depending on the necroptotic stimulus, and the reproducible effect of EFTUD2 previously observed during MRV infection. Once again, we observed variations in the total level of MLKL (Figure 3D), although there was no statistically significant difference when multiple WB were analyzed (Figure 3E). EFTUD2 and SNRNP200 are thus required for induction of necroptosis during MRV infection while EFTUD2’s importance for necroptosis appears to be generalized to other necroptotic stimuli, such as zVAD-fmk/TNFα.

### 3.4. KD of U5 snRNP Core Components Impacts mRNA Levels, Protein Levels, and Alternative Splicing of Necroptotic Regulators

**Figure 4:**
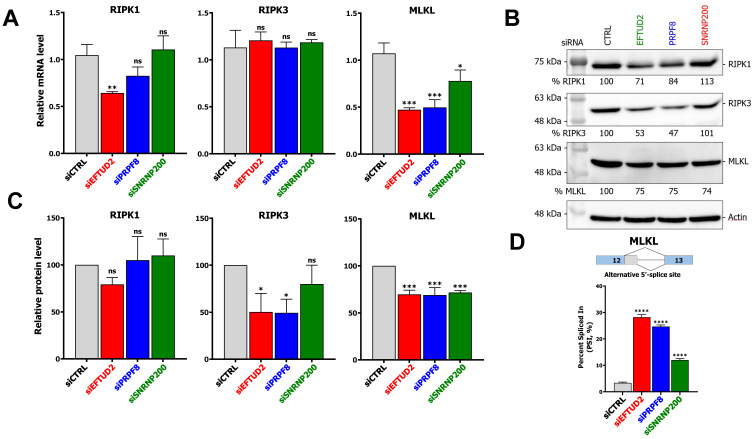
Depletion of U5 snRNP core components impacts the mRNA level, protein levels, and alternative splicing of necroptotic regulators. (**A**) Relative mRNA levels for RIPK1, RIPK3, and MLKL in control or EFTUD2, PRPF8, or SNRNP200-depleted L929 cells as determined by qPCR. L929 cells were transfected with the appropriate siRNA for 72 h before RNA was harvested, reverse transcribed, and subjected to qPCR. PSMC4, PUM1, and TXNL4B were used as housekeeping genes for normalization. n = 3, biological replicates, one-way ANOVA with Dunnett’s multiple comparisons test against the control siRNA condition (ns, *p* > 0.05; *, *p* ≤ 0.05; **, *p* ≤ 0.01; ***, *p* ≤ 0.001). (**B**) Western blot of the main necroptotic regulators (RIPK1, RIPK3, MLKL) in L929 cells treated with control or EFTUD2, PRPF8, or SNRNP200 specific siRNA. Cells were incubated for 72 h after transfection before proteins were harvested for Western blot. Actin was used as a loading control. Relative quantitation is shown compared to the siCTRL condition. (**C**) Quantification and statistical analysis of three independent Western blot for RIPK1, RIPK3 and MLKL. n = 3, biological replicates, one-way ANOVA with Dunnett’s multiple comparisons test against the siCTRL treated cells (ns, *p* > 0.05; *, *p* ≤ 0.05; ***, *p* ≤ 0.001). (**D**) Percent Spliced In (PSI) values for the AS event (alternative 5′ splice site) in MLKL upon depletion of EFTUD2, PRPF8, and SNRNP200. The AS event is depicted on the top. n = 3, biological replicates, one-way ANOVA with Dunnett’s multiple comparisons test against the control siRNA condition (****, *p* ≤ 0.0001).

The preceding results suggest that EFTUD2 KD is protecting cells against necroptosis from MRV infection and zVAD/TNFα treatment, whereas SNRNP200 KD is only able to block necroptosis arising from MRV infection. To understand the impact of depleting the U5 snRNP core components on necroptosis, we first investigated the impact of these depletions on mRNA levels of the main regulators of necroptosis, i.e., RIPK1, RIPK3, and MLKL (Figure 4A). RIPK3 mRNA levels were unaffected by the KD, while MLKL mRNA levels were significantly decreased by all KD. RIPK1 mRNA levels were only reduced upon EFTUD2 KD, pointing to a specific impact of EFTUD2 depletion on RIPK1 mRNA. Then, we profiled the relative protein levels of RIPK1, RIPK3 and MLKL upon depletion of EFTUD2, PRPF8 and SNRNP200. EFTUD2 and PRPF8 depletion reduced the protein levels of all the necroptotic regulators tested, whereas SNRNP200 depletion only reduced MLKL levels (Figure 4B). Quantification of three independent WB revealed no significant reduction of RIPK1 upon U5 core proteins KD, and a significant reduction of RIPK3 upon both EFTUD2 and PRPF8 KD (Figure 4C). Since there is no change in RIPK3 mRNA level upon EFTUD2 and PRPF8 silencing (Figure 4A), these results suggest EFTUD2 and PRPF8 regulate RIPK3 level post-transcriptionally, such as through alternative splicing or post-translationally. Moreover, there was a significant reduction of MLKL protein levels upon KD of all three proteins (Figure 4C). This was previously observed in other experiments (Figure 3B,E), although not statistically significant in these experiments. These results emphasize that U5 snRNP core components control both the mRNA levels and proteins levels of critical proteins involved in necroptosis. Since the impact of EFTUD2 and PRPF8 KD on RIPK3 and MLKL levels are similar, whereas PRPF8 do not protect from necroptosis, these differences in protein levels cannot solely explain the involvement of EFTUD2 and SNRNP200 in necroptosis, and other mechanisms are likely involved. Finally, we wondered if the alternative splicing of RIPK1, RIPK3, and MLKL might be affected by manipulating the levels of the U5 snRNP core components since recent studies have suggested that the U5 snRNP controls cellular AS [5,16]. The only annotated AS event in MLKL, an alternative 5′ splice site, was strongly regulated by the depletion of all U5 snRNP proteins studied herein (Figure 4D). Two AS events in RIPK1, and two AS events in RIPK3, were also analyzed and revealed not to be the subject of AS under the conditions studied (Supplementary Figure S4). These results establish U5 snRNP proteins EFTUD2, PRPF8, and SNRNP200 as important for the regulation of the mRNA levels, the protein levels, and the AS of key necroptotic proteins RIPK1, RIPK3, and MLKL.

### 3.5. EFTUD2 and PRPF8 Are Required for Optimal Induction of the IFN Response

**Figure 5:**
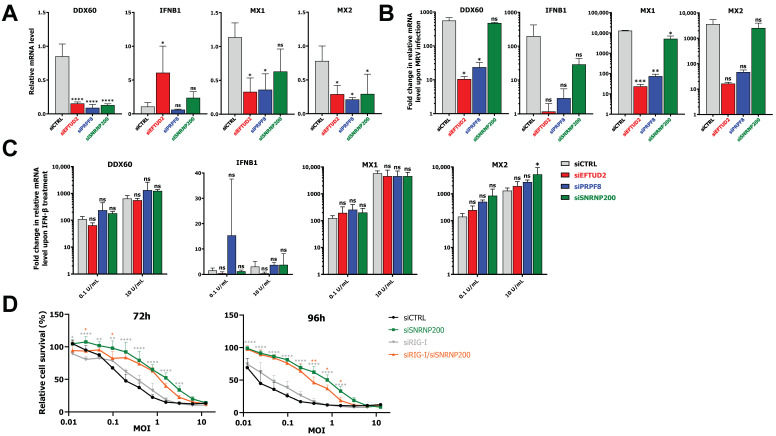
EFTUD2 and PRPF8 control the interferon response during MRV infection. (**A**) Relative mRNA levels for DDX60, IFNB1, MX1, and MX2 in control or EFTUD2, PRPF8, or SNRNP200-depleted L929 cells. L929 cells were transfected with the appropriate siRNA for 72 h before RNA was harvested, reverse transcribed, and subjected to qPCR. PSMC4, PUM1, and TXNL4B were used as housekeeping genes for normalization. n = 3, biological replicates, one-way ANOVA with Dunnett’s multiple comparisons test against the control siRNA condition (ns, *p* > 0.05; *, *p* ≤ 0.05; ****, *p* ≤ 0.0001). (**B**) Relative fold induction upon MRV infection for DDX60, IFNB1, MX1, and MX2 in control or EFTUD2, PRPF8, or SNRNP200-depleted L929 cells. L929 cells were transfected with the appropriate siRNA for 55 h before being either mock-infected or infected with MRV at a MOI of 3 for 24 h. Each replicate was arbitrarily attributed an uninfected control sample to calculate a fold-change upon infection. n = 3, biological replicates, Brown-Forsythe and Welch one-way ANOVA with Dunnett’s T3 multiple comparisons test against the control siRNA condition (ns, *p* > 0.05; *, *p* ≤ 0.05; **, *p* ≤ 0.01; ***, *p* ≤ 0.001). (**C**) Relative fold induction upon IFN-β treatment for DDX60, IFNB1, MX1, and MX2 in control or EFTUD2, PRPF8, or SNRNP200-depleted L929 cells. L929 cells were transfected with the appropriate siRNA for 72 h, and then IFN-β was added for 5 h before RNA was harvested, reverse transcribed and subjected to qPCR. PSMC4, PUM1, and TXNL4B were used as housekeeping genes for normalization. n = 3, biological replicates, two-way ANOVA with Šídák’s multiple comparisons test against the control siRNA treated with the same IFN-β concentration (ns, *p* > 0.05; *, *p* ≤ 0.05). (**D**) Methylene blue staining of control, SNRNP200, RIG-I, or RIG-I/ SNRNP200-depleted L929 cells infected with 1:1 dilutions of MRV at 72 h or 96 h post-infection. Twice the quantity of siRNA was transfected to perform DKD; in the case of single KD, siCTRL was added to match the total siRNA quantity of the DKD. The quantification of cell-bound methylene blue stain is shown for three independent experiments. n = 3, biological replicates, two-way ANOVA with Dunnett’s multiple comparisons test against the SNRNP200 KD condition (in red; *, *p* ≤ 0.05; **, *p* ≤ 0.01; ***, *p* ≤ 0.001; ****, *p* ≤ 0.0001). If not indicated, results are not statistically significant. The results of the comparison against the control siRNA (in black) are not indicated for clarity purposes.

Since an increasing amount of evidence suggests that some U5 snRNP proteins control the interferon response [50,51,52,53], which is also involved in necroptosis [54], we next investigated whether EFTUD2, PRPF8, and SNRNP200 are required for the IFN response. First, we tested if the reduction of U5 core components impacted the basal mRNA levels of IFN-β and three interferon-stimulated genes (ISG; *DDX60*, *MX1*, *MX2*) by qPCR (Figure 5A). A significant reduction of *DDX60* and *MX2* mRNA levels was observed with all U5-directed siRNA, while *MX1* mRNA was also significantly reduced except in the SNRNP200 depletion. This suggests the U5 snRNP is important for the processing and maturation of ISG, even in the absence of viral infection and IFN signaling. In the case of *IFNB1*, only the EFTUD2 KD significantly impacted its mRNA level. Next, we investigated if U5 snRNP core components are required for the IFN response in the context of MRV infection. Control and EFTUD2-, PRPF8- and SNRNP200-depleted cells were infected with MRV, and RNA was harvested at 24 h post-infection. To correct for the impact of these siRNA on the basal levels of ISG, a fold-change was calculated between infected and mock cells for each siRNA to allow adequate quantification of the induction of these genes during infection without the effect of the siRNA. The depletion of EFTUD2 and PRPF8 significantly reduced the IFN response after MRV infection (Figure 5B). In contrast, SNRNP200 had limited effect on the cell’s ability to respond to infection through the IFN response pathway, highlighting once again the specific and different roles of these proteins belonging to the same snRNP. Although quantitatively different, similar effects on the interferon response were also observed upon infection at a higher MOI (Supplementary Figure S5).

The IFN response pathway is divided in two parts: recognition of viral PAMP and production of IFN, and then signaling through the IFN receptor (IFNAR) and induction of ISG. To address whether the involvement of EFTUD2 and PRPF8 is in the first or second part of the pathway, we repeated the preceding experiment, but this time we stimulated the cells using IFN-β instead of using MRV. By doing so, the first part of the pathway is bypassed, and only the IFNAR signaling and ISG induction are involved. First, we treated L929 cells with 10-fold dilutions of recombinant IFN-β and measured the induction of ISG to determine the right concentration of IFN-β to use (Supplementary Figure S6). None of the IFN-β concentrations tested could induce the *IFNB1* gene, highlighting that the observed effect is strictly driven by the exogenous IFN-β added to the medium. Based on these titration curves, we selected 10 U/mL of IFN-β, since this concentration was inducing the maximal effect, and 0.1 U/mL, a concentration sufficient to induce ISG a little less than a 100-fold. Control and EFTUD2-, PRPF8- and SNRNP200-depleted cells were then treated with IFN-β, and RNA was harvested 5 h later. In the absence of EFTUD2, PRPF8, and SNRNP200, the fold induction of *DDX60*, *MX1,* and *MX2* was not significantly reduced in comparison to the control condition (Figure 5C). This supports the idea that the impact previously observed in MRV-infected cells (Figure 5B) of EFTUD2 and PRPF8 is not on the second part of the pathway (signaling through IFNAR and induction of ISGs), but rather lies before, probably during recognition of viral PAMP and production of IFN.

Having shown that SNRNP200 had little effect on IFN induction during MRV infection, we further validated that its impact on cell survival is not dependent on the interferon pathway. To do so, we depleted RIG-I, one of the principal cytoplasmic sensors of viral dsRNA and ssRNA during MRV infection that allows for the production of primary IFN and subsequent induction of ISG [55,56]. The depletion of RIG-I in L929 using siRNA was previously shown to be highly efficient [5] and validated again using qPCR (Supplementary Figure S1). We depleted RIG-I and SNRNP200 individually, or in combination, and looked at overall cell survival using methylene blue staining as before. The DKD is slightly reduced compared to KD of SNRN200, but still presents an overall increased survival compared to the single RIG-I KD (Figure 5D). We thus conclude that only a marginal part of the effect of SNRNP200 is mediated by the IFN response pathway, as KD of RIG-I minimally decreases its protective effect. Similar experiments were performed for EFTUD2; however, the interpretation of the results was much more complex since the survival induced by EFTUD2 is much closer to the control condition than SNRNP200 in this assay (Supplementary Figure S7). From these experiments, we conclude that both EFTUD2 and PRPF8 control the IFN response during MRV infection, and SNRNP200′s requirement for cell death during MRV infection is mainly not IFN-mediated.

### 3.6. EFTUD2 Restricts MRV’s Replication in Both Single Cycle and Multiple Cycles of Replication

**Figure 6:**
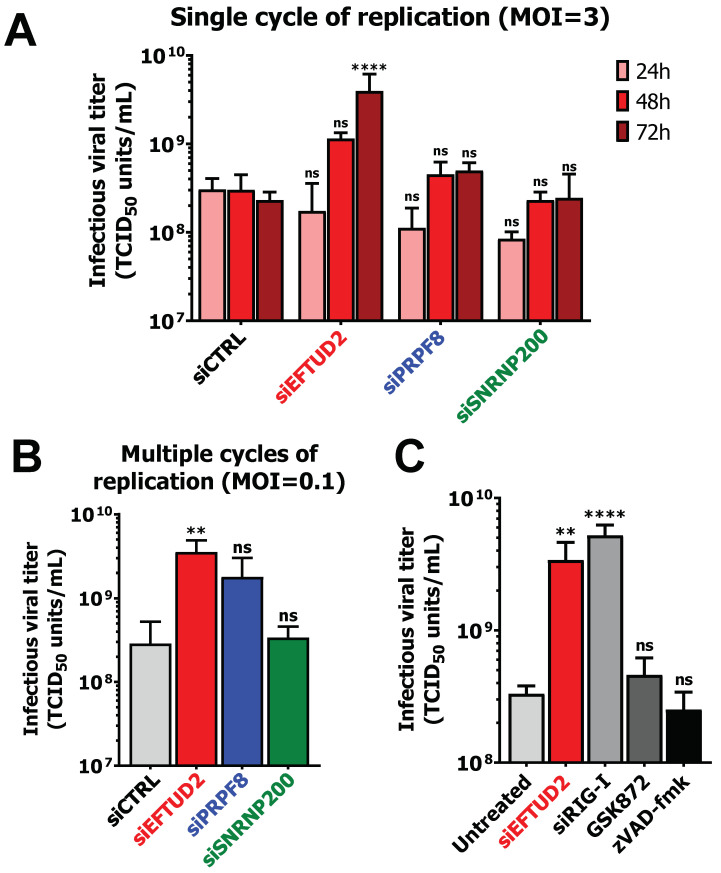
EFTUD2 restricts MRV’s replication in both single cycle and multiple cycles of replication. (**A**) MRV viral titers from siCTRL, siEFTUD2, siPRPF8, and siSNRNP200-treated L929 cells infected cells at 24 h, 48 h, and 72 h. Cells were infected at a MOI of 3, and viral titers were determined by TCID_50_ on L929 cells. n = 3, biological replicates, two-way ANOVA with Dunnett’s multiple comparisons test against the siCTRL matched timepoint (ns, *p* > 0.05; ****, *p* ≤ 0.0001). (**B**) MRV viral titers from siCTRL, siEFTUD2, siPRPF8 and siSNRNP200-treated L929 cells infected cells at 96 h. Cells were infected at a MOI of 0.1, and viral titers were determined by TCID_50_ on L929 cells. n = 3, biological replicates, one-way ANOVA with Dunnett’s multiple comparisons test against the siCTRL condition (ns, *p* > 0.05; **, *p* ≤ 0.01). (**C**) MRV viral titers in L929 cells untreated, transfected with a siRNA against EFTUD2 or RIG-I, or treated with GSK872 (3 μM) or zVAD-fmk (50 μM) to block necroptosis and apoptosis, respectively. L929 cells were infected at a MOI of 3, and viral titers were determined by TCID_50_ on L929 cells at 72 h post-infection. n = 3, biological replicates, one-way ANOVA with Dunnett’s multiple comparisons test against the untreated condition (ns, *p* > 0.05; **, *p* ≤ 0.01; ****, *p* ≤ 0.0001).

The results presented herein highlight novel roles of U5 snRNP proteins EFTUD2, PRPF8, and SNRNP200 in apoptosis, necroptosis, and interferon induction, using MRV as a model for viral infection. However, one last question remains to this point: are these U5 snRNP proteins antiviral? Since they are involved in critical aspects of the battle between the virus and the cell (i.e., the interferon response and cell death), one could hypothesize that they indeed contribute to limit viral replication, since they are required for cell death and induction of the interferon response. To answer this question, we infected L929 cells with MRV at a MOI of 3 upon KD of EFTUD2, PRPF8, and SNRNP200 and followed viral replication by TCID_50_ at 24 h, 48 h and 72 h. In the control siRNA condition, maximal viral replication was already obtained at 24 h (Figure 6A), as expected during MRV’s replication cycle [57]. When PRPF8 or SNRNP200 were depleted, no increase in viral replication could be detected at these time points (Figure 6A). However, EFTUD2 KD significantly enhanced the replication at 72 h by 10-fold (Figure 6A). Since EFTUD2-depleted cells survive longer after infection (Figure 1A,C,D,E), this increased survival is also accompanied by prolonged viral replication beyond the normal replication cycle. This effect is also specific for EFTUD2, since there is no increase in viral replication in SNRNP200-depleted cells that also present the cell-survival phenotype (Figure 1A,C,D,E). We next wanted to assess if the same increase in viral replication is maintained during multiple replication cycles, since EFTUD2-depleted cells could potentially exert a protective effect by keeping the virus intracellular and preventing its release through increased survival. L929 cells were thus infected with MRV at a MOI of 0.1 upon KD of EFTUD2, PRPF8, and SNRNP200, and viral replication was quantified at 96 h. Once again, only the EFTUD2 KD enhanced viral replication significantly by 10-fold (Figure 6B). Since the same increase is observed in both single cycle and multiple replication cycles, we conclude that EFTUD2 KD does not block the release of MRV from infected cells beyond the normal replication cycle. Furthermore, it also suggests that the virus might not be released by cytolysis but by a non-cytolytic pathway since cell survival is increased, as suggested recently for reovirus and other members of the *reoviridae* family [58,59,60]. Moreover, EFTUD2 restricts MRV replication, both in a single cycle and multiple cycles of replication. We previously showed that EFTUD2 is required for apoptosis (Figure 2D), necroptosis (Figure 3A,B,E,F), and efficient interferon response (Figure 5B) during MRV infection; we finally wondered if the antiviral effect of EFTUD2 is mediated through its role in apoptosis, necroptosis, or the interferon response. First, we assessed the best way to chemically block necroptosis during MRV infection by using GSK963 (targeting RIPK1) or GKS872 (targeting RIPK3) (Supplementary Figure S8). GSK872 could completely abrogate MLKL phosphorylation in MRV-infected cells; however, GSK963 could only reduce the levels of p-MLKL by approximately 50%. We thus utilized GSK872 to block necroptosis, zVAD-fmk to block apoptosis, and a siRNA against RIG-I to limit the interferon response and monitored viral replication at 72 h to decipher the respective role of apoptosis, necroptosis and the interferon response on MRV replication. Both zVAD-fmk and GSK872 did not significantly impact MRV’s viral titers, suggesting that apoptosis and necroptosis have little influence on MRV replication (Figure 6C). However, limiting the interferon response using a siRNA against RIG-I significantly enhanced MRV replication, in a range similar to the EFTUD2 depletion. This result suggests the antiviral activity of EFTUD2 is principally mediated by its impact on the interferon response, and minimally by its role in apoptosis and necroptosis.

## 4. Discussion

**Figure 7:**
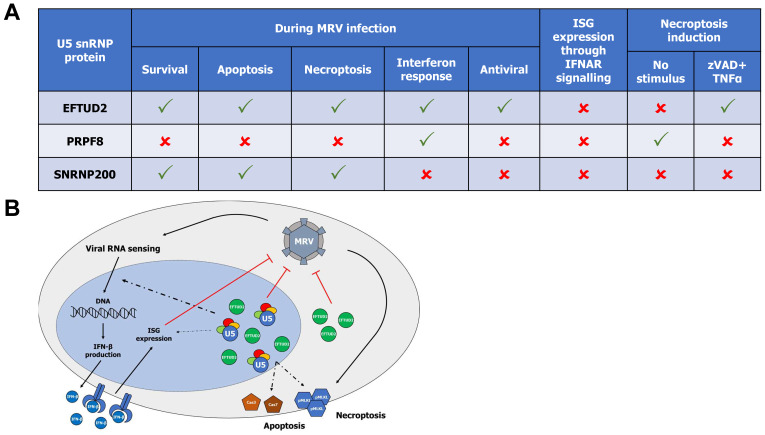
Summary of the involvement of U5 snRNP core proteins in virus–host interactions. (**A**) Table depicting the respective roles of EFTUD2, PRPF8, and SNRNP200 in the interferon response pathway, apoptosis, and necroptosis. (**B**) Model depicting the roles of EFTUD2 during MRV infection. EFTUD2 (either cytoplasmic or nuclear; and either in the U5 snRNP or by itself when located in the nucleus) exerts an antiviral role on MRV replication (red arrow). MRV infection elicits both apoptosis and necroptosis (black arrow), which require the presence of EFTUD2 to normally proceed (dotted arrow). EFTUD2 is also required for the basal expression of ISG (thin dotted arrow), but its principal impact on the IFN response pathway during infection lies somewhere between the sensing of viral RNA and the production of IFN (dotted arrow). ISG expression exerts an antiviral effect on MRV (red arrow), and might explains, at least in part, the antiviral effect of EFTUD2, since MRV actively trigger this pathway through the sensing of its viral RNA (black arrow).

In this study, we demonstrated distinct and overlapping crucial roles of U5 snRNP core components EFTUD2, PRPF8, and SNRNP200 on apoptosis, necroptosis, and interferon induction during viral infection, using MRV as a model virus. We demonstrated that EFTUD2 and SNRNP200 are required for both apoptosis and necroptosis, whereas EFTUD2 and PRPF8 are required for optimal interferon response against viral infection. These results are summarized in Figure 7A. Previous studies had already hinted at the role of U5 snRNP core components in apoptosis. Expression of pathogenic PRPF8 variants responsible for retinitis pigmentosa induced apoptosis [61]. Retinitis pigmentosa is a genetic disorder of the eyes characterized by a loss of vision and is sometimes caused by mutations in specific spliceosomal proteins, such as PRPF8 and SNRNP200. Moreover, increased PRPF8 protein levels in ovarian cancer cells protect cells from apoptosis [62]. Our results support these findings, as depleting PRPF8 led to enhanced annexin V staining (Figure 2B) and enhanced cell death (Figure 2C) in uninfected cells. However, we did not observe any defect in cell proliferation by RTCA (Figure 1B), any difference in cell death by FACS (Figure 1C), nor any significant enhancement in Cas3/7 activity when silencing PRPF8 in the absence of infection (Figure 2D). It thus indicates that the effect of PRPF8 on apoptosis is minor, at least in L929 cells, and might be challenging to observe, especially if cells are already dead by the time the assay is performed. Other studies have demonstrated that EFTUD2 performs a similar role, where depletion of EFTUD2 leads to enhanced apoptosis in developmental models and cancer cell lines [63,64,65]. EFTUD2 depletion also impacts the splicing of mRNA encoding survival and cell death proteins, suggesting that the involvement of EFTUD2 in apoptosis might be, at least in part, splicing-dependent [66]. Our results do not point to any impact of EFTUD2 depletion on apoptosis in the absence of MRV infection in L929 cells (Figure 1B,C and Figure 2B–D). The impact of EFTUD2 on apoptosis might depend on other determinants, such as the status of p53, as previously demonstrated by others [64,66]. Further research should clarify the involvement of EFTUD2 and PRPF8 in apoptosis in different cellular contexts, both in the absence and presence of apoptosis triggers, and the determinants that might influence these roles.

Since the link between apoptosis and some U5 snRNP core components had already been established, we focused our experiments on necroptosis, which had never been previously linked to U5 proteins. Once again, both EFTUD2 and SNRNP200 protected L929 cells from MRV-induced necroptosis. Moreover, EFTUD2 also protected from zVAD/TNFα-mediated necroptosis, whereas with this stimulus, SNRNP200 exerted no protective effect, and even sensitized cells to necroptosis (Figure 3C,D,E). The opposite effects of SNRNP200 on necroptosis based on the stimulus triggering necroptosis is interesting; determining how SNRNP200 protects cells from MRV-mediated necroptosis will help understand why during zVAD/TNFα treatment necroptosis is enhanced in the absence of SNRNP200, whereas its depletion protects against MRV-mediated necroptosis. Once again, we observed the induction of cell death, this time necroptosis, when PRPF8 is depleted (Figure 2A,B), very similarly to apoptosis. However, this effect is not always observed, notably during zVAD/TNFα treatment (Figure 3D). The difference between these two experiments is the time allowed to accumulate dead cells in the medium; in Figure 3A, cells were harvested 40 h after infection, a very long incubation time that could lead to a significant accumulation of dead cells in the medium. In contrast, zVAD/TNFα treatment is much shorter (4 h), and cells are rinsed before zVAD-fmk pretreatment. This thus suggests that the number of necroptotic cells when PRPF8 is depleted is low and requires long incubation times to accumulate sufficiently for detection. Nevertheless, the observation that depletion of PRPF8 triggers necroptosis raises further important questions. Which cells are actively undergoing necroptosis? Are these the ones that have the most drastic reduction of PRPF8? And what is sensed in those cells as the trigger for necroptosis, and through which sensor? This raises the interesting hypothesis that misspliced RNA might somehow be sensed and trigger necroptosis, or at least could be involved in necroptosis regulation. Available interactome data for MLKL, RIPK1, and RIPK3 seem to support this hypothesis, as MLKL interactors are enriched for RNA binding protein and splicing factors as well as for nuclear proteins, which is not the case for RIPK1 and RIPK3 (Supplementary Figure S9). Notably, six proteins from the U5 snRNP (DDX23, EFTUD2, PRPF6, PRPF8, SNRNP40, and SNRNP200) are amongst the interactors of MLKL, suggesting that these U5 snRNP proteins, either alone or assembled in the mature snRNP, might interact with MLKL, and point to some U5-driven regulation of necroptosis. The importance of nuclear-driven phenomenon during necroptosis further hints at a nuclear component of its regulation [67,68,69]. During apoptosis, some caspases, such as caspase-7, are notably regulated through RNA to target RNA binding proteins, further emphasizing the critical role of RNA in the regulation of programmed cell death [70,71].

Both apoptosis and necroptosis are triggered during MRV infection [42,45]. However, the roles of these respective programmed cell death pathways and how they are intertwined have not been systemically deciphered. For rotavirus, a closely related pathogenic virus causing diarrhea in infants, recent work revealed that apoptosis and necroptosis are balanced together, and when one cell death pathway is chemically blocked, the other takes over [72,73]. The absence of cell death is detrimental during multiple viral replication cycles, supporting the vision that cell death allows for the release of the progeny virions from the host cell and viral spread [72]. However, in a single replication cycle, the effects of both necroptosis and apoptosis are less clear; a study showed no impact of necroptosis on viral replication [72], whereas another suggested apoptosis is antiviral and necroptosis is proviral [73]. MRV’s determinants of apoptosis have been well-studied, with the involvement of viral proteins σ1, σ3, μ1, and possibly σ1s [22,42,74,75]. However, determinants of MRV-mediated necroptosis are much less known and require further studies. Viral replication is required, and chemically blocking RIPK1 seems to reduce MRV-driven necroptosis [44,45]. In this study, we observed that blocking RIPK1 using GSK963 could not completely abrogate MLKL phosphorylation, whereas blocking RIPK3 using GSK872 efficiently suppresses all phosphorylation of MLKL (Supplementary Figure S8). This result suggests that MRV-induced necroptosis is not strictly sensed by RIPK1, and that other necroptotic sensors might be involved. MRV’s cell entry is notably endosomal, where another necroptotic sensor, TRL3, is located [21,22]. TRL3 recognizes dsRNA, such as that present in MRV’s internal capsid and recently shown to be exposed during entry and thus could likely trigger necroptosis from the endosome by sensing MRV’s dsRNA [76,77]. The role of TLR3 in MRV’s infection requires further studies, as its involvement is still unclear [78,79,80,81]. More research should also be performed to determine if MRV’s proteins are directly triggering necroptosis. In the case of rotavirus, NSP4 is directly responsible for both apoptosis and necroptosis [73]. We have shown previously that MRV’s μ2 protein interacts with EFTUD2, PRPF8, and SNRNP200, and that these proteins are required for MRV’s impact on cellular AS [5,23]. A polymorphism linked to the impact on AS, P208S, had no impact on the ability of μ2 to interact with these U5 snRNP [5]. We now demonstrate that some of these proteins are required for induction of necroptosis. Whether the μ2 protein might be involved in the induction of necroptosis during MRV infection warrants further studies.

We also demonstrated that the silencing of EFTUD2 and PRPF8 significantly limits both interferon-β and ISG induction during MRV infection (Figure 5B). These results support other studies that have suggested a role of U5 proteins in the interferon pathway [50,51,52,53]. Indeed, both EFTUD2 and SNRNP200 have been shown to sense viral RNA in the cytoplasm, setting off the interferon response [51,52]. Furthermore, EFTUD2 also controls the AS of *MYD88*, a key player of the signal transduction upstream IFN production [53]. However, in MRV-infected L929 cells, SNRNP200 exerted no control over the IFN response, whereas PRPF8 had an effect very similar to EFTUD2, albeit to a lesser extent. Further studies should clarify if these discrepancies are cell line specific or arise through an indirect effect of one of the KD. It is notably important to underline that the MRV strain we used in this study, T3D^S^, has previously been shown to induce low levels of IFN and to have a low sensitivity to its effect, as compared to the reverse genetics virus, T3D^K^ [26]. It would be interesting to replicate these experiments with T3D^K^ and confirm if an increased effect can be observed when using a MRV strain that induces more IFN and is more sensitive to its effect. Nonetheless, the aforementioned mechanism of viral RNA sensing in the cytoplasm of U5 snRNP core components was also confirmed in this study, as directly treating cells with IFN-β completely abrogated EFTUD2 and PRPF8 control of the IFN response. This raises the interesting question as to where in the cell is the U5 snRNP and/or the U5 components controlling the IFN response. Are individual components in the cytoplasm having this second role before their loading in the mature U5 snRNP and import into the nucleus, as shown previously for EFTUD2 and SNRNP200 [51,52]? Is this role linked to the mature U5 snRNP and AS-dependent, as it was demonstrated for MYD88 [53]? Finally, we cannot rule out that the U5 snRNP complex fully assembled in the nucleus might control the induction of IFN and ISG not linked to AS. All these possibilities warrant further studies into the role of the U5 snRNP in the IFN response. Finally, we also demonstrated that EFTUD2 restrict MRV’s replication, both in a single cycle and multiple cycles of replication. Neither blocking apoptosis or necroptosis alone could enhance MRV’s titers, but limiting the IFN response could enhance MRV’s titer to levels similar to the depletion of EFTUD2, suggesting EFTUD2 exerts its antiviral activity mainly by its role in the IFN pathway. EFTUD2′s roles during MRV infection are summarized in Figure 7B.

We previously demonstrated that MRV reduces the protein levels of EFTUD2, PRPF8, and SNRNP200 through the action of the μ2 protein during infection [5]. We now show a direct benefit for MRV to induce this reduction, which allows for enhanced cell survival through reduced apoptosis and necroptosis, diminished IFN response, and enhanced viral replication. As U5 snRNP emerged recently as a targeted cellular component during viral infection [4,5,6], the study presented herein further strengthens our understanding of the benefit for viruses to destabilize the U5 snRNP through its crucial role in regulating apoptosis, necroptosis, and the IFN response pathway.

## Data Availability

Not applicable.

## Supplementary Materials

The following supporting information can be downloaded at: https://www.mdpi.com/article/10.3390/v14122710/s1. Figure S1: qPCR validation of the knock-down of EFTUD2, PRPF8, SNRNP200 and RIG-I (DDX58); Figure S2: RTCA of T3DS-infected L929 cells transfected with a control siRNA or EFTUD2, PRPF8, or SNRNP200 specific siRNA; Figure S3: Live cell imaging of control or EFTUD2, PRPF8, or SNRNP200-depleted L929 cells treated with zVAD/TNF-α to induce necroptosis; Figure S4: AS-PCR for the AS events in RIPK1 and RIPK3 upon EFTUD2, PRPF8 and SNRNP200 depletion; Figure S5: Relative fold induction upon MRV infection for DDX60, IFNB1, MX1, and MX2 in control or EFTUD2, PRPF8, or SNRNP200-depleted L929 cells; Figure S6: Relative mRNA levels for DDX60, IFNB1, MX1, and MX2 in L929 cells treated with logarithmic dilutions of IFN-β; Figure S7: Methylene blue staining of control, EFTUD2, RIG-I, or RIG-I/EFTUD2- depleted L929 cells infected with binary dilutions of T3DS at 72 h or 96 h post- infection; Figure S8: Western blot of phosphorylated MLKL (pMLKL) and total MLKL in mock- or T3DS-infected cells treated with necroptosis inhibitors (GSK872, GSK963) or apoptosis inhibitor (zVAD-fmk); Figure S9: Gene ontology (GO) analysis of the protein interactors for key necroptotic regulators RIPK1, RIPK3 and MLKL; Figure S10: Design maps for the ASE analyzed by AS-PCR in L929 cells; Figure S11: Uncropped western blots from this study; Table S1: qPCR primers; Table S2: AS-PCR primers. Refs. [82,83] cited in Supplementary Materials file.
