# Supplementary material for: U5 snRNP Core Proteins Are Key Components of the Defense Response against Viral Infection through Their Roles in Programmed Cell Death and Interferon Induction
Source: Viruses. 2022 Dec 3;14(12):2710. doi: 10.3390/v14122710 (PMC9785106; doi:10.3390/v14122710)
Supplement: Supplementary file 1 [file viruses-14-02710-s001.zip › viruses-2036398-supplementary.pdf]

Supplementary Figures

U5 snRNP core proteins are key components of the defense response against viral infection through their roles in programmed cell death and interferon induction

Simon Boudreault, Guy Lemay, Martin Bisaillon

Table S1. qPCR primers

Target	Housekeeping gene	Species	Primer	
			Fw	Rv
RIPK1		Mouse	5′-GCTTTGGCATTGTCCTTTGGGC-3′	5′-GGCTGATGATCTCCCTTGGACAG-3′
RIPK3		Mouse	5′-TACACAGCTTGAACCCTCCGCT-3′	5′-ACCCTCCCTGAAACGTGGACAG-3′
MLKL		Mouse	5′-TGTCAGCCAGCCAGCATCCT-3′	5′-GGGTTTTGTTGATTTCTTCCACGCT-3′
DDX60		Mouse	5′-CCACCACAGTTCCATGAGTGCC-3′	5′-TGATTCCCAAAGCGAGTGTTCCA-3′
IFNB1		Mouse	5′-ACACTGCCTTTGCCATCCAAGA-3′	5′-ACACTGTCTGCTGGTGGAGTTCA-3′
MX1		Mouse	5′-GAAGGAGAGGAGTGGAGAGGCA-3′	5′-GCTTATCACTGATCCCCAGGCC-3′
MX2		Mouse	5′-GGAAGCTGAGGAGGAGAAGAAGACA-3′	5′-GCTGGAGATGCGGTTGTGAGC-3′
DDX58		Mouse	5′-AACTTGCTTTGGAGAAAGACAACAGC-3′	5′-CGCCTCCGCTCCATCATCCT-3′
EFTUD2		Mouse	5′-TCGGCTCAGTGAAGGACAGCAT-3′	5′-GGGCAACCACAGCATCCAGT-3′
PRPF8		Mouse	5′-CCACCAGCCTTTGAGAGACAGTAG-3′	5′-GGCCAGTCGGTAGAGTGTGGAC-3′
SNRNP200		Mouse	5′-AACTGGAGCGAGAGGAGGAAGT-3′	5′-TGAGGCTGTTGGACTTGGCGT-3′
PSMC4	✓	Mouse	5′-CCCAGGAGGAGGTGAAGCGG-3′	5′-GGTCGATGGTACTCAGGATGCG-3′
PUM1	✓	Mouse	5′-TGCCAGTCTCTTCCAGCAGCA-3′	5′-TGATTTGGGGTCAAAGGACGTTGG-3′
TXNL4B	✓	Mouse	5′-CCCTCTACCGTATTTTTCTTCAATGGGC-3′	5′-AGTTTTCCCCTCATCGCTCCCC-3′

Table S2. AS-PCR primers

Gene	Primers		ASE present in this region	Expected peak(s) (bp)	Detected peak(s) (bp)	ASE quantified	Corresponding detected peak(s) (bp)
	Fw	Rv					
MLKL	5′-CAGGATTGCCCTGAGTTGTTGC-3′	5′-CCTCTTACACCTTCTTGTCCGT-3′	5′-SS	136, 160	136, 160, 261, 269	5′-SS	136, 160
RIPK1	5′-GGAACTATTCGCTGGTGATGGA-3′	5′-TGTCATTCAGGTGTTCGGGTGC-3′	Exon cassette	210, 348	348, 471	Exon cassette	348
RIPK1	5′-GCCCGTCCTTCCTCACCTAG-3′	5′-AACACAAGGACACCTTCCCGA-3′	Possible exon cassette of the alternative first exon	212, 292	212	Exon cassette	212
RIPK3	5′-CTCCCGACGATGTCTTCTGTCA-3′	5′-ACTCCGAACCCTCCTTTACCCA-3′	3′-SS	96, 113, 194	113, 382	3′-SS	113
RIPK3	5′-TTGAACCCTCCGCTCCTGCA-3′	5′-CAGCCAGCACTGCCCACACG-3′	Exon cassette	104, 262	262	Exon cassette	262

Figure S1. qPCR validation of the knock-down of EFTUD2, PRPF8, SNRNP200 and RIG-I (DDX58). L929 were transfected with the appropriate siRNA for 72 h before RNA was harvested, reverse transcribed and subjected to qPCR. *PSMC4*, *PUM1*, and *TXNL4B* were used as housekeeping genes for normalization. n=3, biological replicates, one-way ANOVA with Dunnett's multiple comparisons test against the control siRNA condition for EFTUD2, PRPF8, and SNRNP200; unpaired two-tailed Student’s t-test for RIG-I (ns, P>0.05; **, P≤0.01; ***, P≤0.001; ****, P≤0.0001).

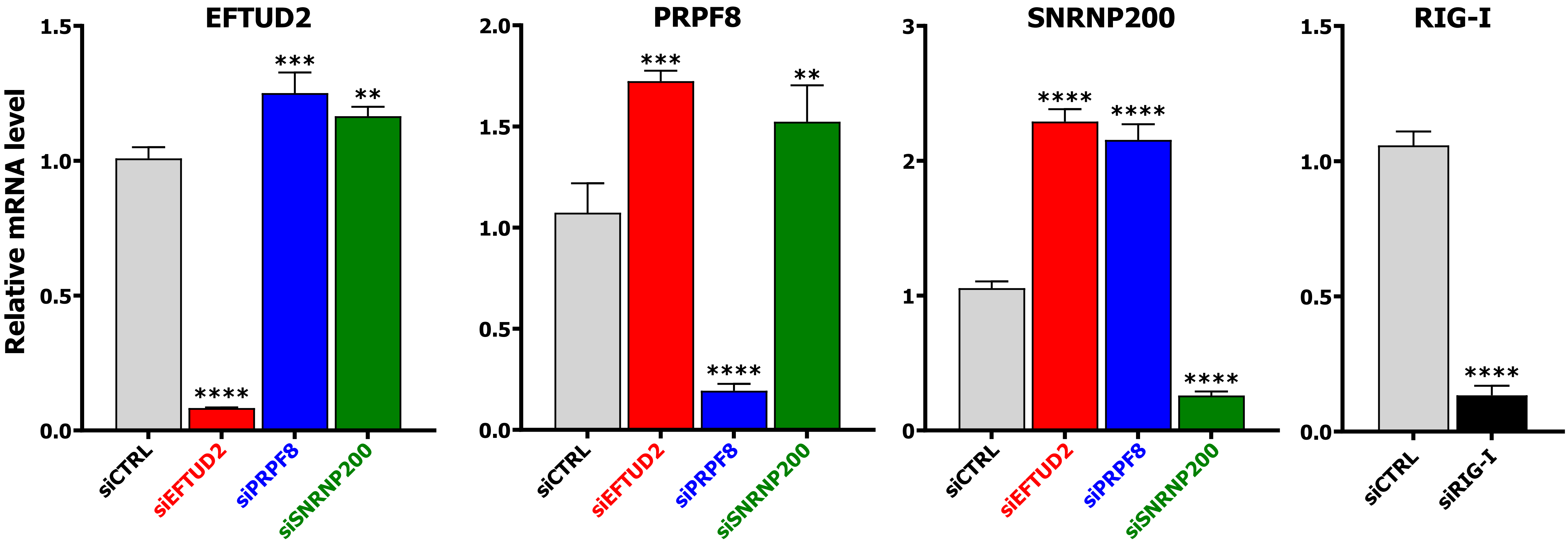

EFTUD2
PRPF8
SNRNP200
RIG-I
Relative mRNA level
1.5
1.0
0.5
0.0
2.0
3
2
1
0
***
**
****
siCTRL
siEFTUD2
siPRPF8
siSNRNP200
siRIG-I

Figure S1

Figure S2. RTCA of T3D^S-infected L929 cells transfected with a control siRNA or EFTUD2, PRPF8, or SNRNP200 specific siRNA. L929 cells were seeded at 5000 cells/well in E-plates and transfected the next morning with the appropriate siRNA. 55 h later, cells were infected at a MOI of 50 and monitored using the xCELLigence system. The relative cell index was normalized to the 1 h 20 min timepoint after the monitoring resumed, because the monitoring in the first hour post-infection was not stable. Measurements were taken every 10 min for up to 90 h after infection; the graph was cut at 60h because lysis was already complete by that timepoint. n=3, biological replicates.

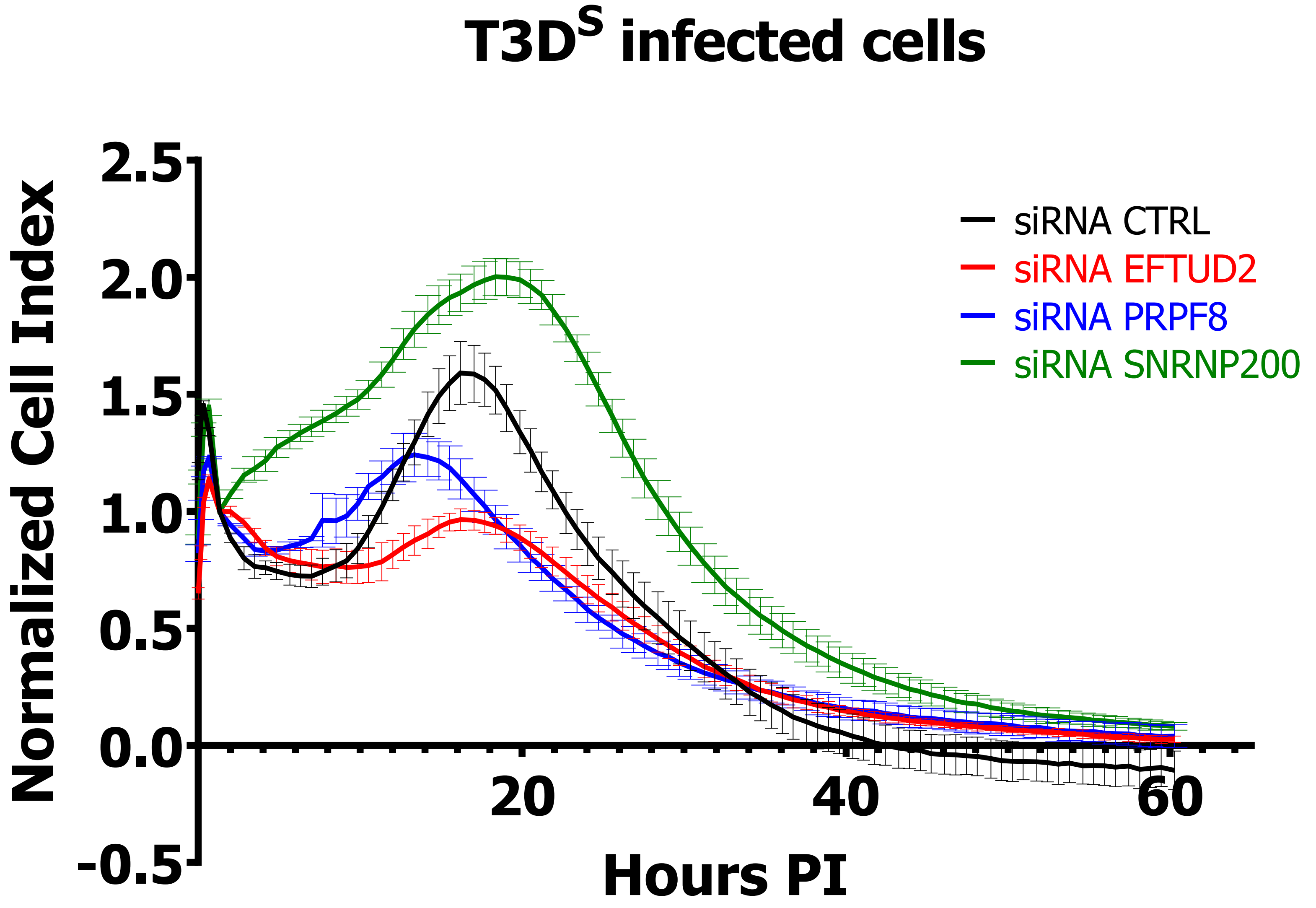

T3D^S infected cells
Normalized Cell Index
2.5
2.0
1.5
1.0
0.5
0.0
-0.5
20
40
60
Hours PI
siRNA CTRL
siRNA EFTUD2
siRNA PRPF8
siRNA SNRNP200

Figure S2

Figure S3. Live cell imaging of control or EFTUD2, PRPF8, or SNRNP200-depleted L929 cells treated with zVAD/TNF-α to induce necroptosis. Cells were transfected with the appropriate siRNA for 72 h. Then, they were pretreated for 1h with zVAD-fmk (100 μM) before TNF-α (25 ng/mL) was added directly to the culture medium. Cells were then directly imaged using a Nikon TE2000E epifluorescence microscope using the differential interference contrast (DIC) channel, gain at 100, 4x objective and a 40 ms exposure time for a period of three days. The black scale bars represent 100 μm.

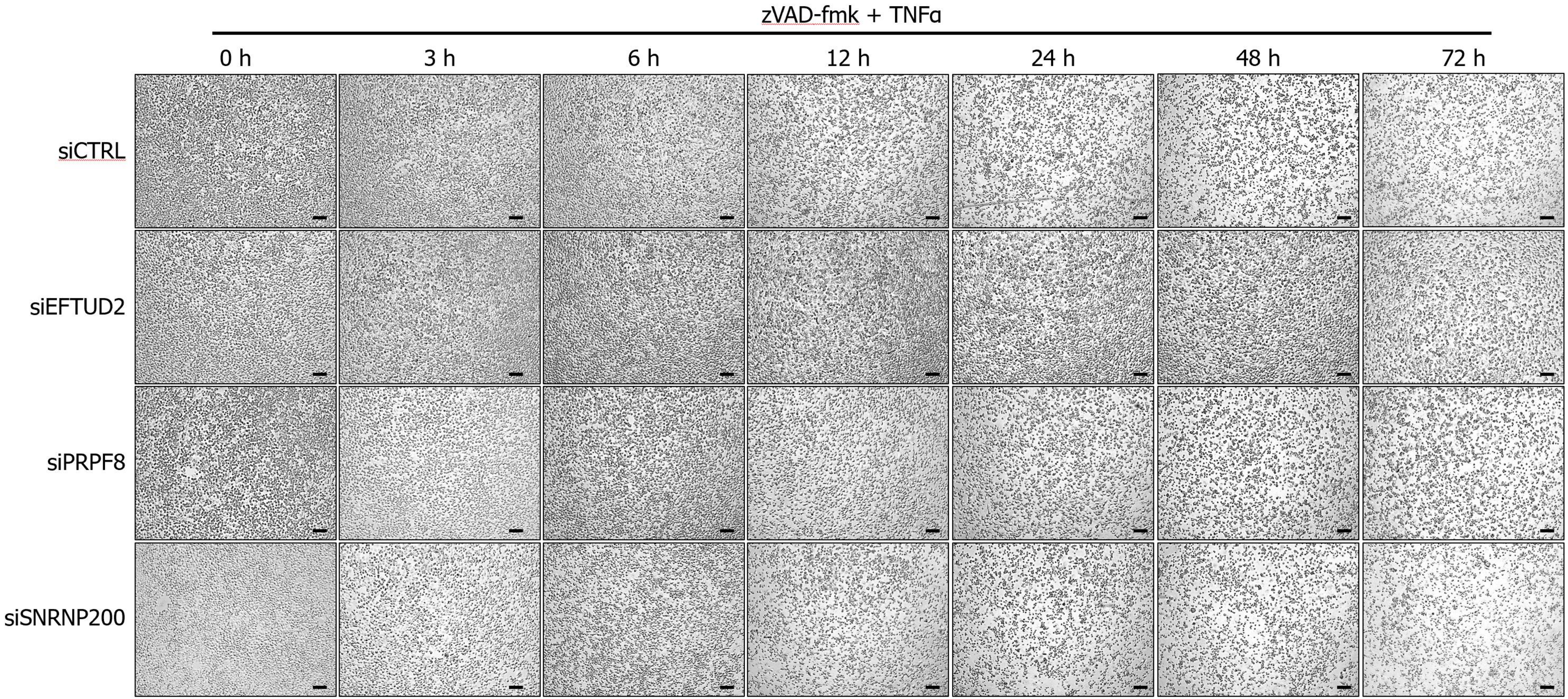
zVAD-fmk + TNFα
0 h
3 h
6 h
12 h
24 h
48 h
72 h
siCTRL
siEFTUD2
siPRPF8
siSNRNP200

Figure S3

Figure S4. AS-PCR for the AS events in RIPK1 and RIPK3 upon EFTUD2, PRPF8 and SNRNP200 depletion. Percent Spliced In (PSI) values for the two AS event in RIPK1 and the two AS events in RIPK3 upon depletion of EFTUD2, PRPF8 and SNRNP200. The AS events are depicted on the top of the graph. n=3, biological replicates.

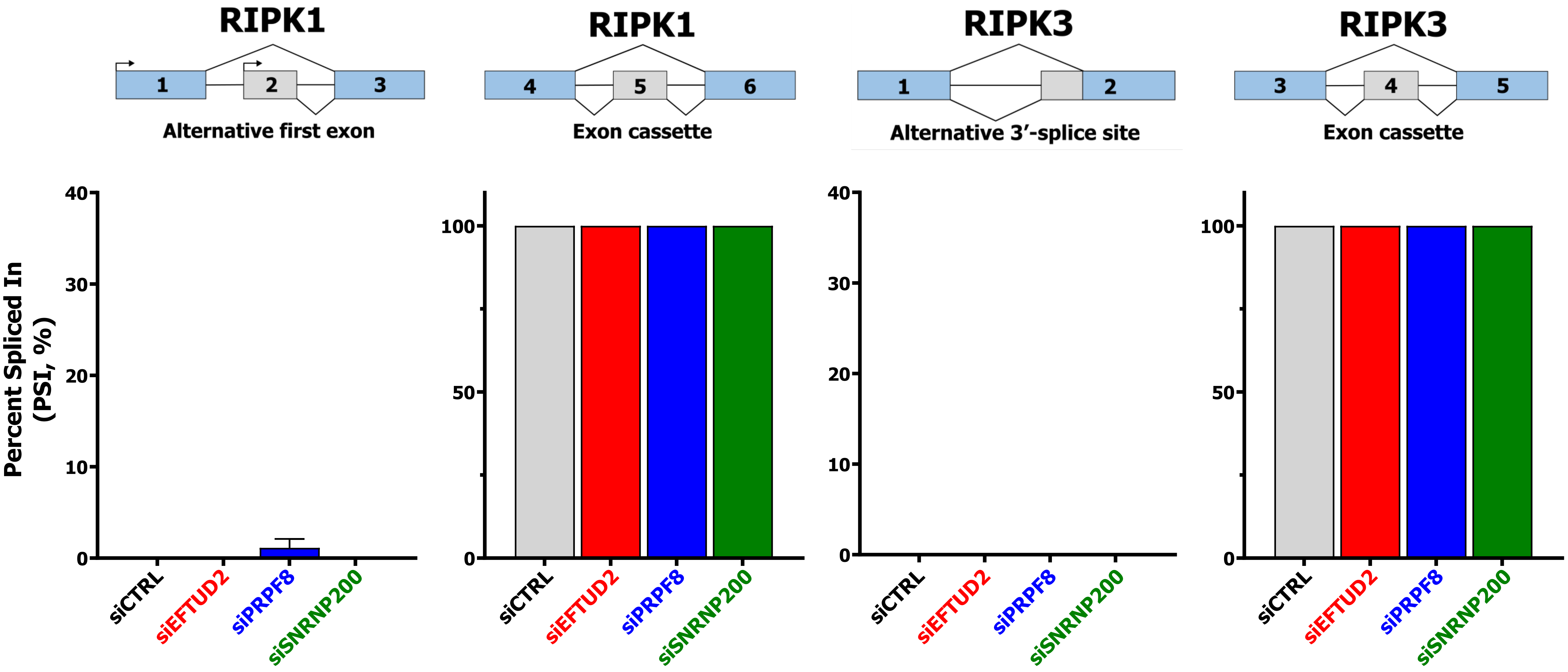
RIPK1
1
2
3
Alternative first exon
RIPK1
4
5
6
Exon cassette
RIPK3
1
2
Alternative 3′-splice site
RIPK3
3
4
5
Exon cassette
Percent Spliced In
(PSI, %)
40
30
20
10
0
100
50
0
40
30
20
10
0
100
50
0
siCTRL
siEFTUD2
siPRPF8
siSNRNP200

Figure S4

Figure S5. Relative fold induction upon MRV infection for *DDX60*, *IFNB1*, *MX1*, and *MX2* in control or EFTUD2, PRPF8, or SNRNP200-depleted L929 cells. L929 were transfected with the appropriate siRNA for 55 h before being either mock-infected or infected with T3D^{S} at a MOI of 50 for 16 h. RNA was then harvested, reverse transcribed and subjected to qPCR. *PSMC4*, *PUM1*, and *TXNL4B* were used as housekeeping genes for normalization. Each replicate was arbitrarily attributed an uninfected control sample to calculate a fold-change upon infection. Since the variance ratio was superior to 1.5, Brown-Forsythe and Welch one-way ANOVA with Dunnett's T3 multiple comparisons test against the control siRNA condition was used for every comparison. The only exception was the comparison between siPRPF8 and siCTRL for IFNB1, where the variance ratio was inferior to 1.5 and one-way ANOVA with Dunnett's multiple comparisons test against the control siRNA condition was applied. n=3, biological replicates (ns, $P>0.05$; **, $P\leq0.01$; ***, $P\leq0.001$).

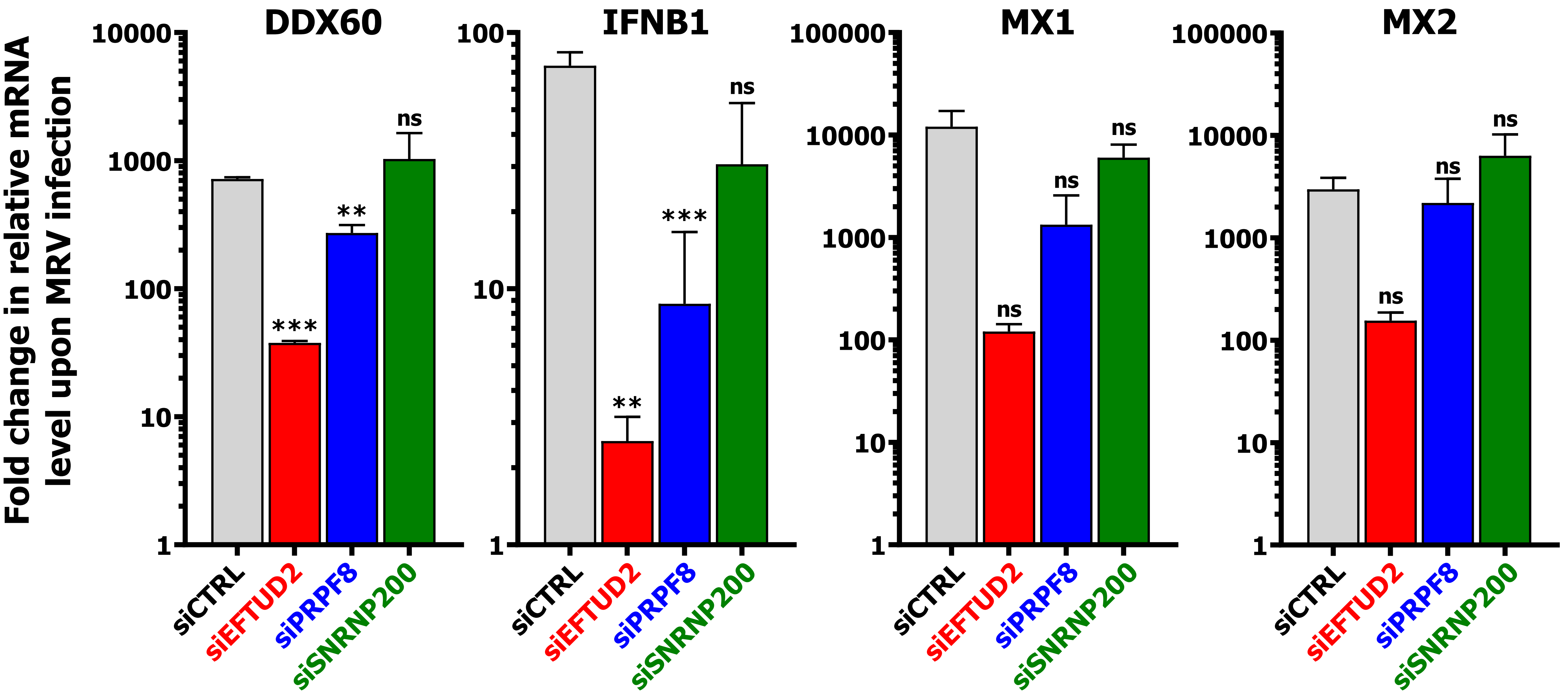
Fold change in relative mRNA level upon MRV infection
DDX60
IFNB1
MX1
MX2
10000
1000
100
10
1
100
10
1
100000
10000
1000
100
10
1
100000
10000
1000
100
10
1
***
**
ns
**
***
ns
ns
ns
ns
ns
ns
ns
siCTRL
siEFTUD2
siPRPF8
siSNRNP200

Figure S5

Figure S6. Relative mRNA levels for *DDX60*, *IFNB1*, *MX1*, and *MX2* in L929 cells treated with logarithmic dilutions of IFN-β. L929 were transfected with a control siRNA for 72 h before IFN-β (0.0001 U/mL to 1000 U/mL) was added for 5 h. RNA was then harvested, reverse transcribed and subjected to qPCR. *PSMC4*, *PUM1*, and *TXNL4B* were used as housekeeping genes for normalization. n=1, technical triplicates.

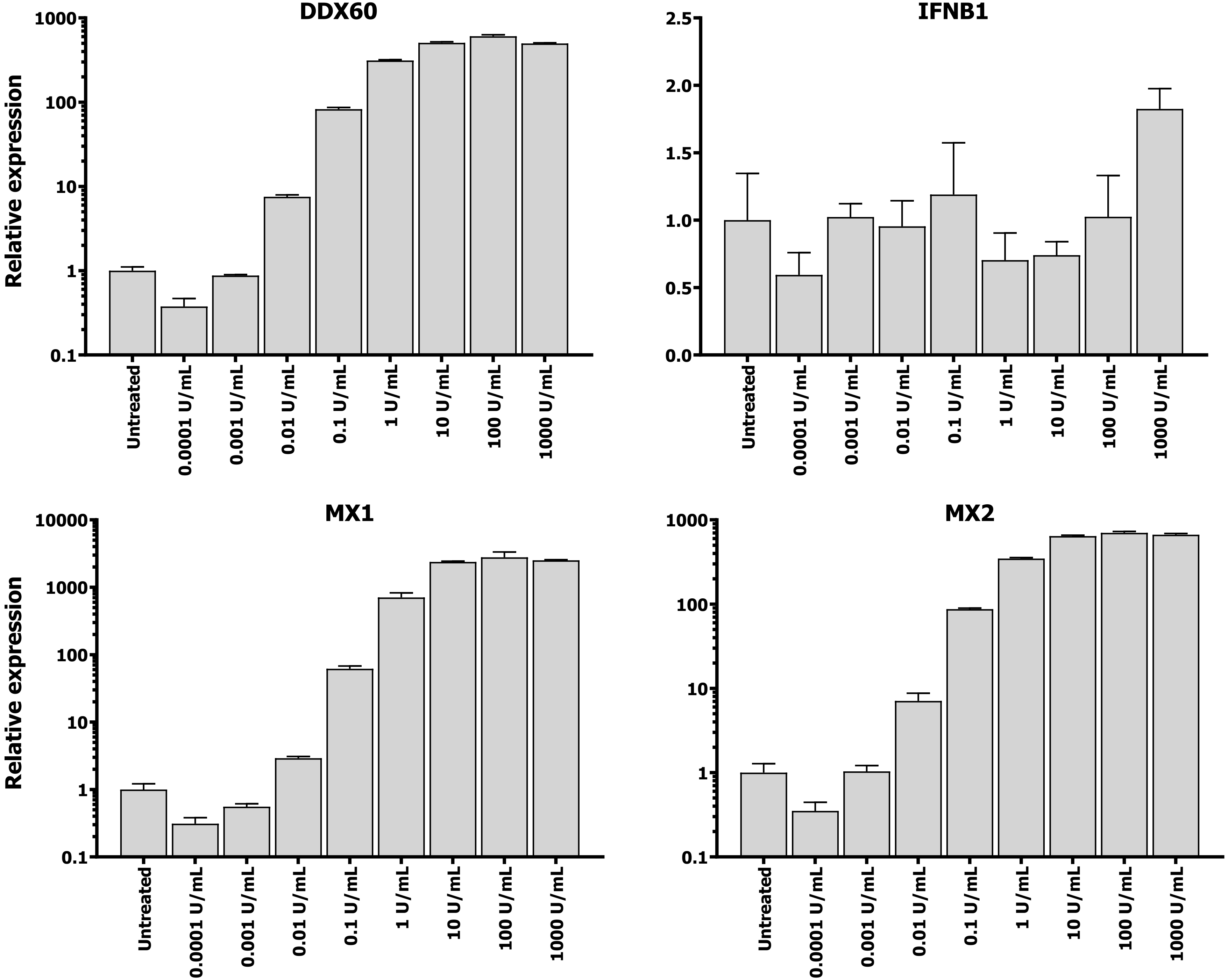
DDX60
IFNB1
MX1
MX2
Relative expression
Relative expression
1000
100
10
1
0.1
2.5
2.0
1.5
1.0
0.5
0.0
10000
1000
100
10
1
0.1
1000
100
10
1
0.1
Untreated
0.0001 U/mL
0.001 U/mL
0.01 U/mL
0.1 U/mL
1 U/mL
10 U/mL
100 U/mL
1000 U/mL

Figure S6

Figure S7. Methylene blue staining of control, EFTUD2, RIG-I, or RIG-I/EFTUD2-depleted L929 cells infected with binary dilutions of $T3D^S$ at 72 h or 96 h post-infection. Twice the quantity of siRNA was transfected to perform the DKD; in the case of a single KD, siCTRL was added to match the total siRNA quantity of the DKD. The quantification of cell-bound methylene blue stain is shown for three independent experiments. n=3, biological replicates, two-way ANOVA with Dunnett's multiple comparisons test against the EFTUD2 KD condition (in red; *, P≤0.05; **, P≤0.01). If not indicated, results are not statistically significant. The results of the comparison against the control siRNA (in black) are not indicated for clarity purpose.

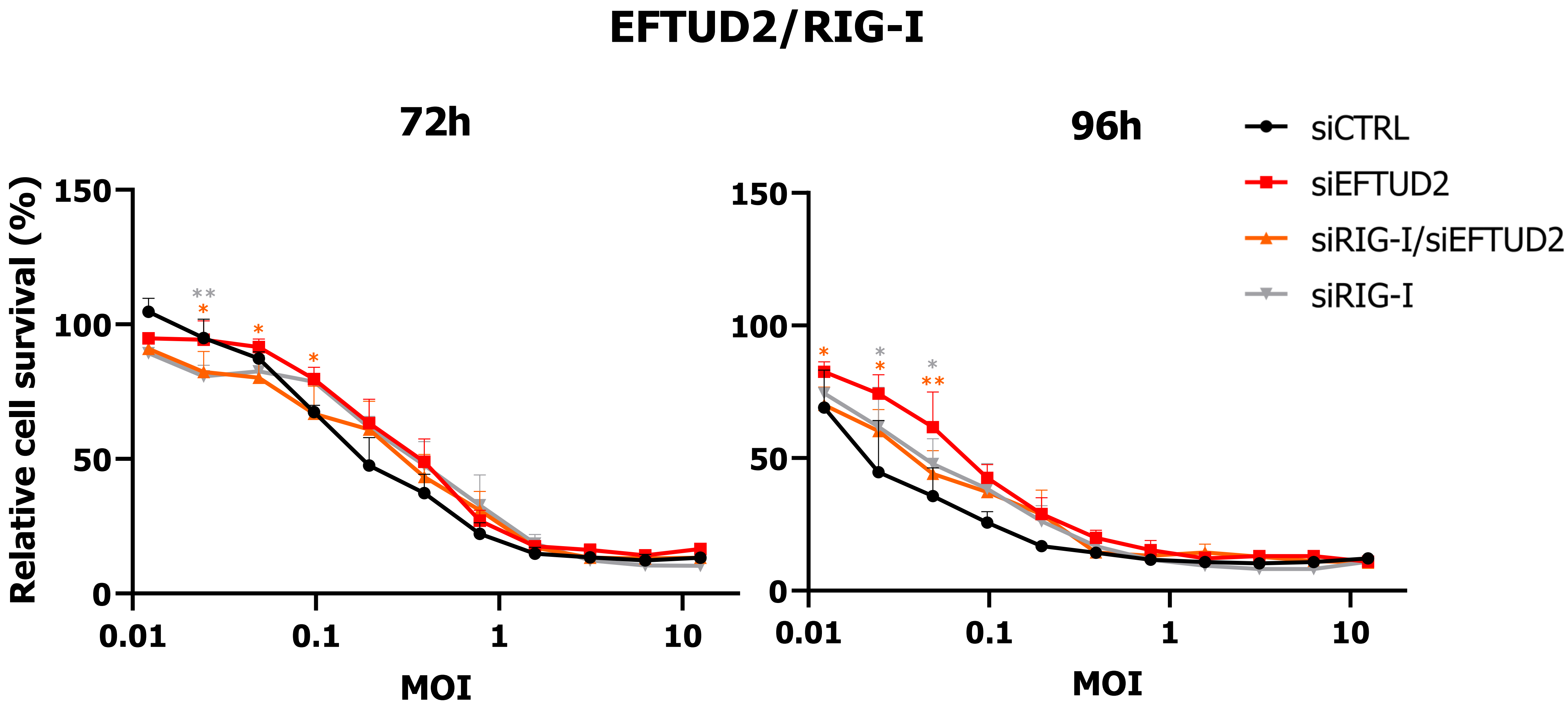

EFTUD2/RIG-I
72h
96h
Relative cell survival (%)
150
100
50
0
0.01
0.1
1
10
MOI
siCTRL
siEFTUD2
siRIG-I/siEFTUD2
siRIG-I

Figure S7

Figure S8. Western blot of phosphorylated MLKL (pMLKL) and total MLKL in mock- or T3D^{S}-infected cells treated with necroptosis inhibitors (GSK872, GSK963) or apoptosis inhibitor (zVAD-fmk). L929 cells were infected at an MOI of 3 and left untreated or treated with the RIPK3 inhibitor GSK872 (3 μM), the RIPK1 inhibitor GSK963 (3 μM) or the pan-caspase inhibitor zVAD-fmk (100 μM) for 40 h before proteins were harvested. Relative quantitation is shown for pMLKL/MLKL.

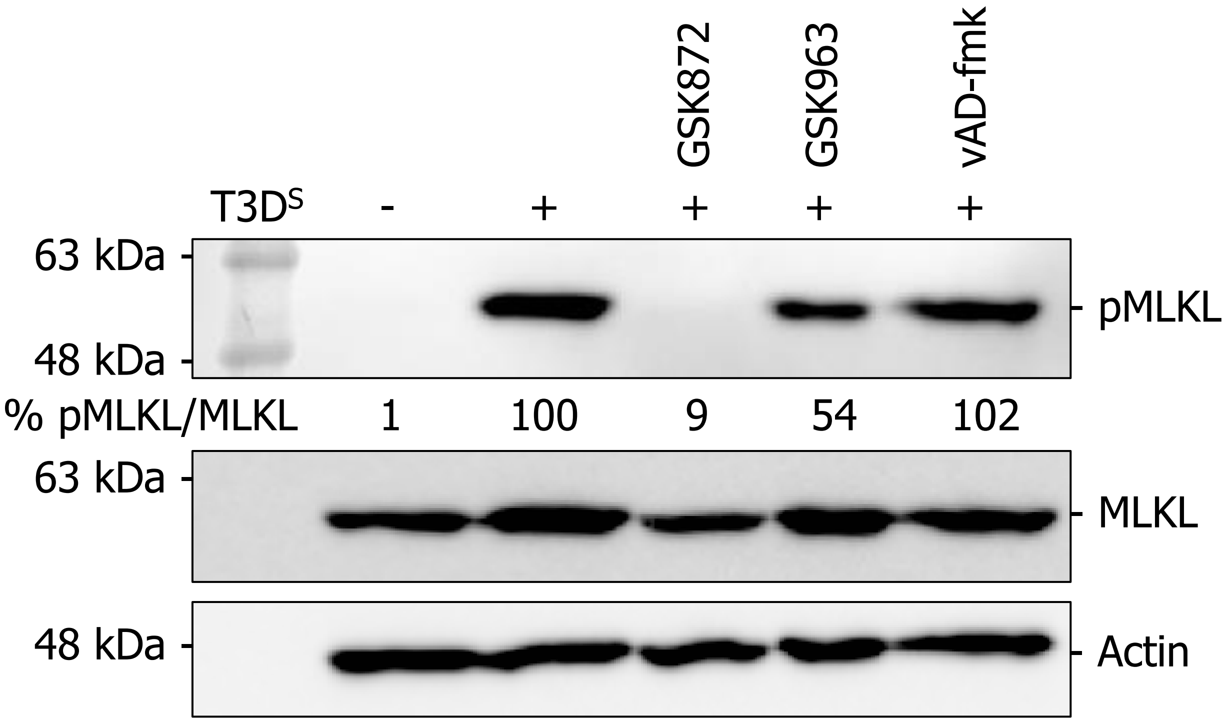
GSK872
GSK963
vAD-fmk
T3D^S
-
+
+
+
+
63 kDa
48 kDa
pMLKL
% pMLKL/MLKL
1
100
9
54
102
63 kDa
MLKL
48 kDa
Actin

Figure S8

Figure S9. Gene ontology (GO) analysis of the protein interactors for key necroptotic regulators RIPK1, RIPK3 and MLKL. Protein interactors were retreived from the Harmonizome database[1] (RIPK1, 137 interactors; RIPK3, 112 interactors; MLKL, 537 interactors). The gene ontology analysis of the interactors was performed using DAVID web server[2] with the gene ontology (biological process or cellular compartment) direct function. The complete list of human genes was used as the background. The Bonferroni correction was used to correct for multiple statistical tests.

[1]Rouillard, A. D. *et al.* The harmonizome: a collection of processed datasets gathered to serve and mine knowledge about genes and proteins. *Database* 2016, baw100 (2016).

[2]Sherman, B. T. *et al.* DAVID: a web server for functional enrichment analysis and functional annotation of gene lists (2021 update). *Nucleic Acids Research* gkac194 (2022) doi:10.1093/nar/gkac194.

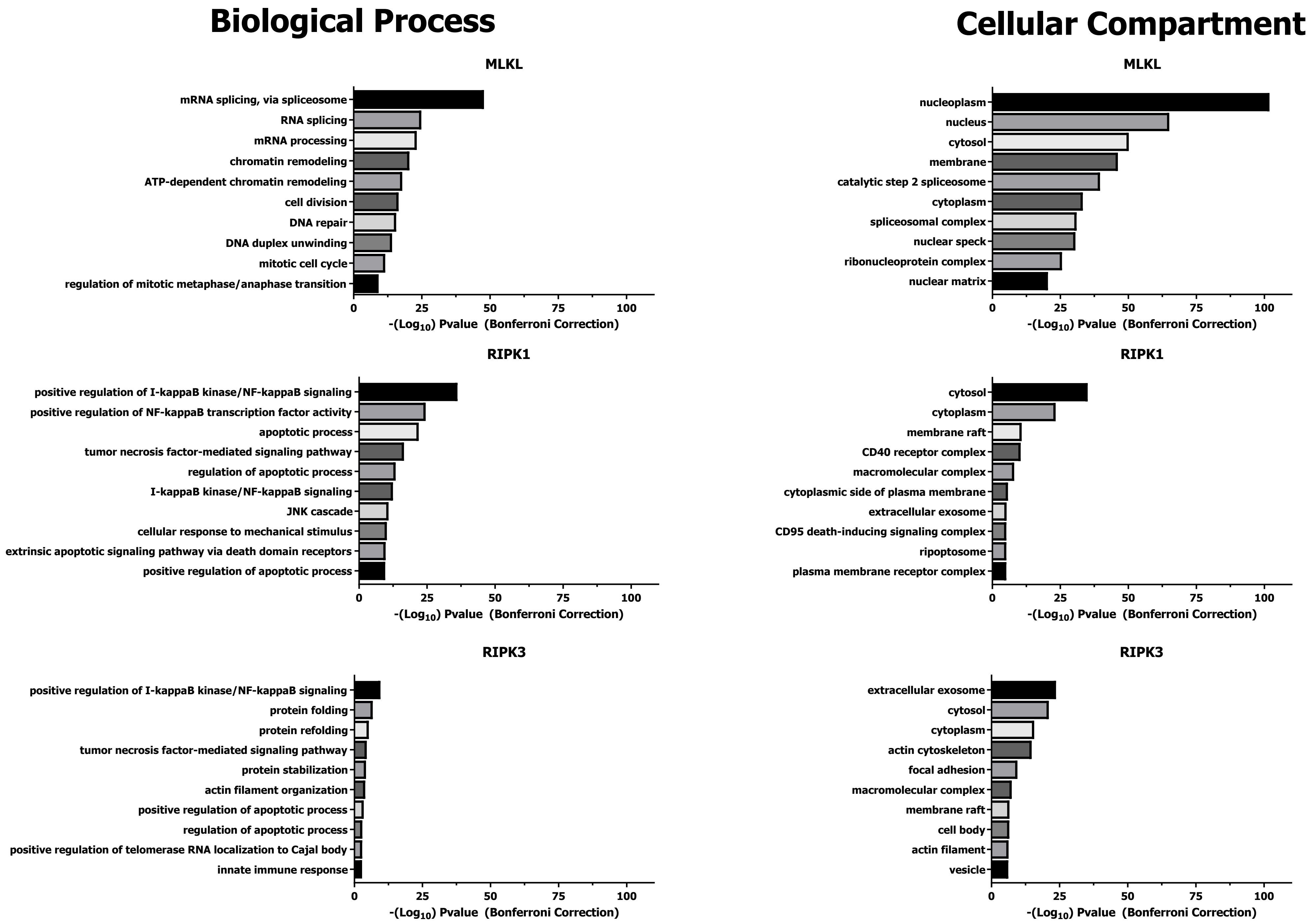

Biological Process
Cellular Compartment
MLKL
mRNA splicing, via spliceosome
RNA splicing
mRNA processing
chromatin remodeling
ATP-dependent chromatin remodeling
cell division
DNA repair
DNA duplex unwinding
mitotic cell cycle
regulation of mitotic metaphase/anaphase transition
0 25 50 75 100
-(Log10) Pvalue (Bonferroni Correction)
MLKL
nucleoplasm
nucleus
cytosol
membrane
catalytic step 2 spliceosome
cytoplasm
spliceosomal complex
nuclear speck
ribonucleoprotein complex
nuclear matrix
0 25 50 75 100
-(Log10) Pvalue (Bonferroni Correction)
RIPK1
positive regulation of I-kappaB kinase/NF-kappaB signaling
positive regulation of NF-kappaB transcription factor activity
apoptotic process
tumor necrosis factor-mediated signaling pathway
regulation of apoptotic process
I-kappaB kinase/NF-kappaB signaling
JNK cascade
cellular response to mechanical stimulus
extrinsic apoptotic signaling pathway via death domain receptors
positive regulation of apoptotic process
0 25 50 75 100
-(Log10) Pvalue (Bonferroni Correction)
RIPK1
cytosol
cytoplasm
membrane raft
CD40 receptor complex
macromolecular complex
cytoplasmic side of plasma membrane
extracellular exosome
CD95 death-inducing signaling complex
ripoptosome
plasma membrane receptor complex
0 25 50 75 100
-(Log10) Pvalue (Bonferroni Correction)
RIPK3
positive regulation of I-kappaB kinase/NF-kappaB signaling
protein folding
protein refolding
tumor necrosis factor-mediated signaling pathway
protein stabilization
actin filament organization
positive regulation of apoptotic process
regulation of apoptotic process
positive regulation of telomerase RNA localization to Cajal body
innate immune response
0 25 50 75 100
-(Log10) Pvalue (Bonferroni Correction)
RIPK3
extracellular exosome
cytosol
cytoplasm
actin cytoskeleton
focal adhesion
macromolecular complex
membrane raft
cell body
actin filament
vesicle
0 25 50 75 100
-(Log10) Pvalue (Bonferroni Correction)

Figure S9

Figure S10. Design maps for the ASE analyzed by AS-PCR in L929 cells. All the RNA isoforms for each gene are depicted alongside their ENSEMBL transcript ID. The primers, designed to amplify the region alternatively spliced, are shown as gray rectangles. On the bottom, a predicted size for the PCR amplicon is given for each transcript. Maps were generated using the mouse assembly GRCm38 (v92) from Ensembl.

MLKL

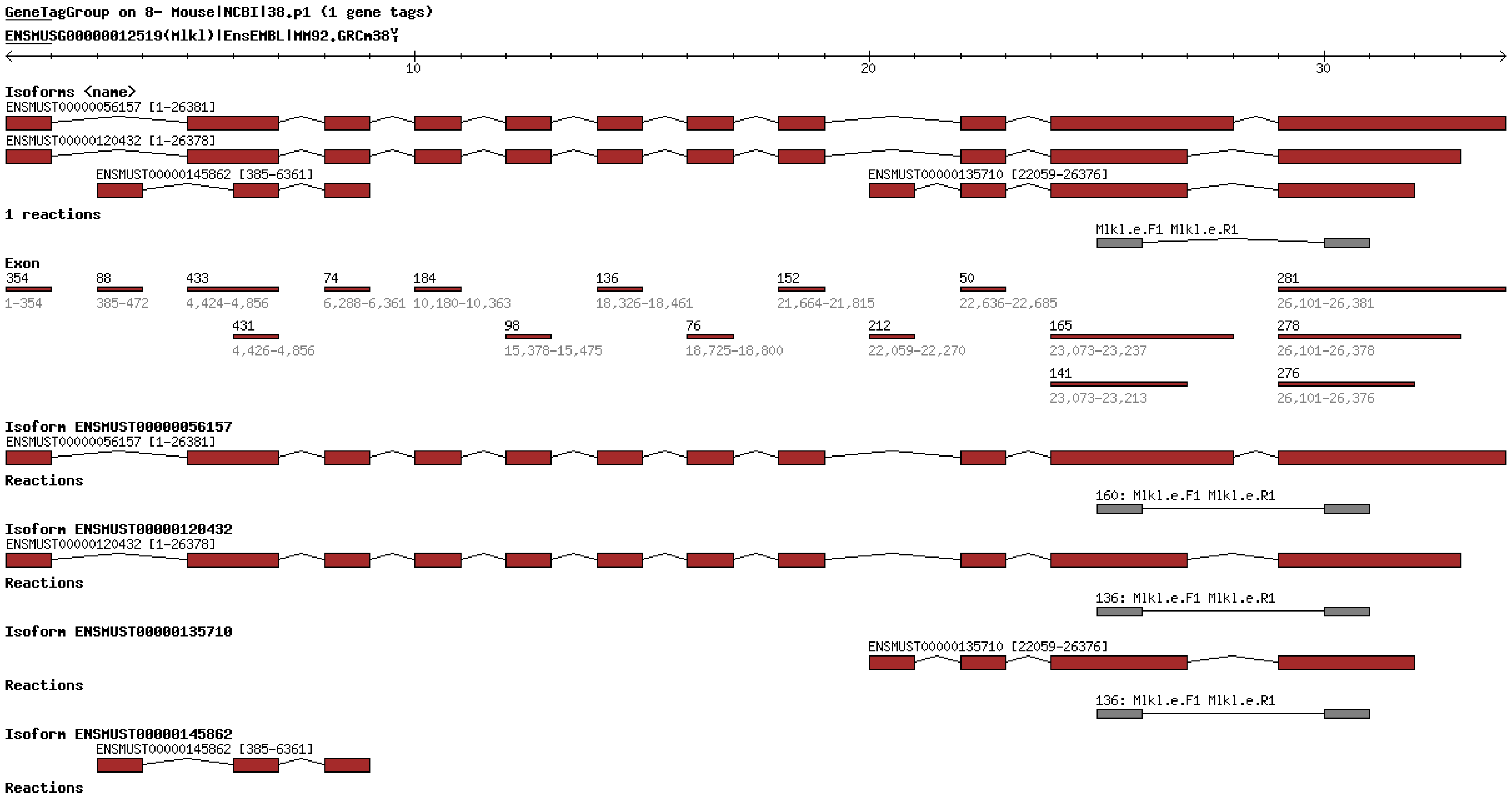

Figure S10

RIPK1

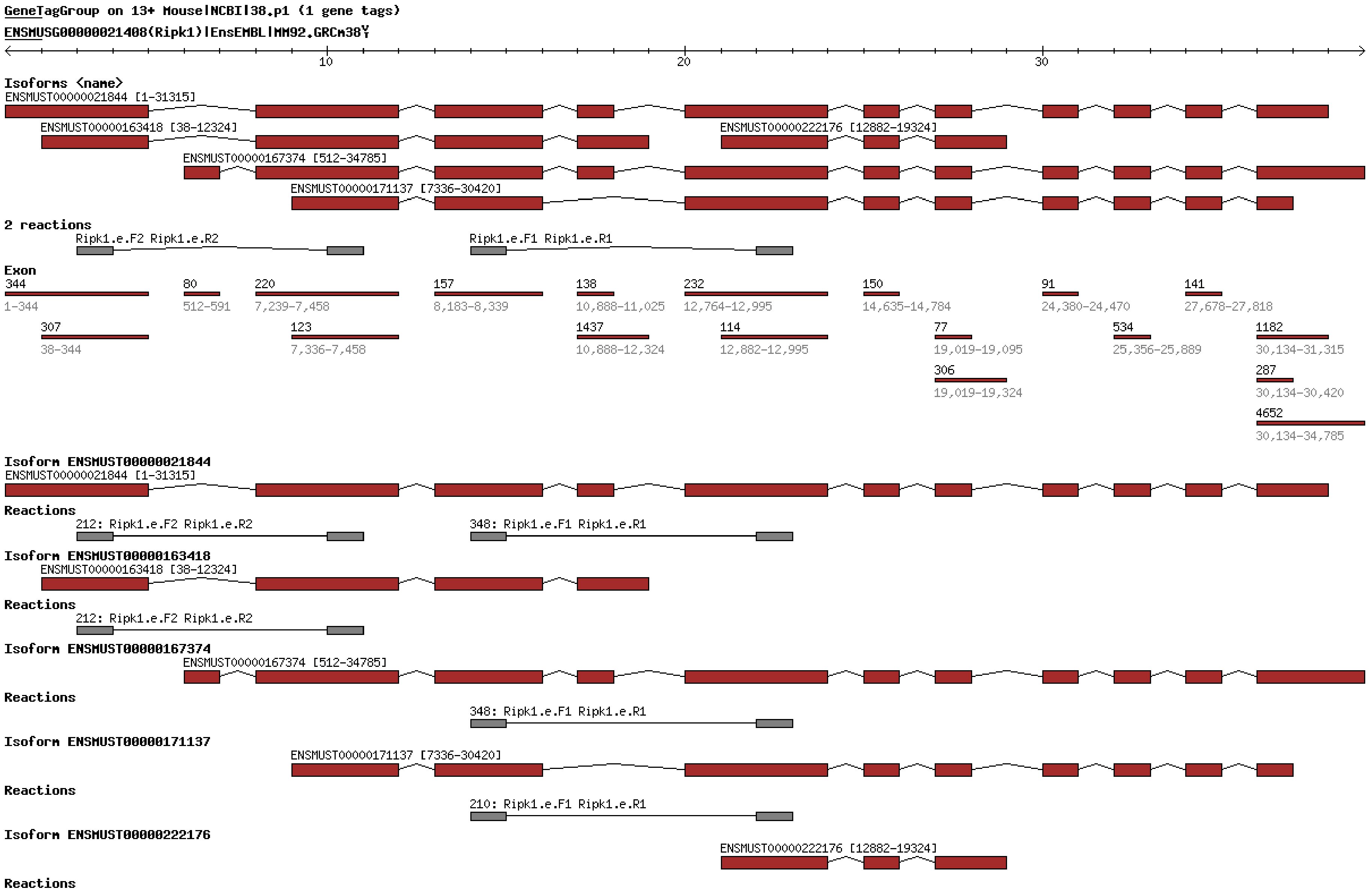

Figure S10 (cont'd)

RIPK3

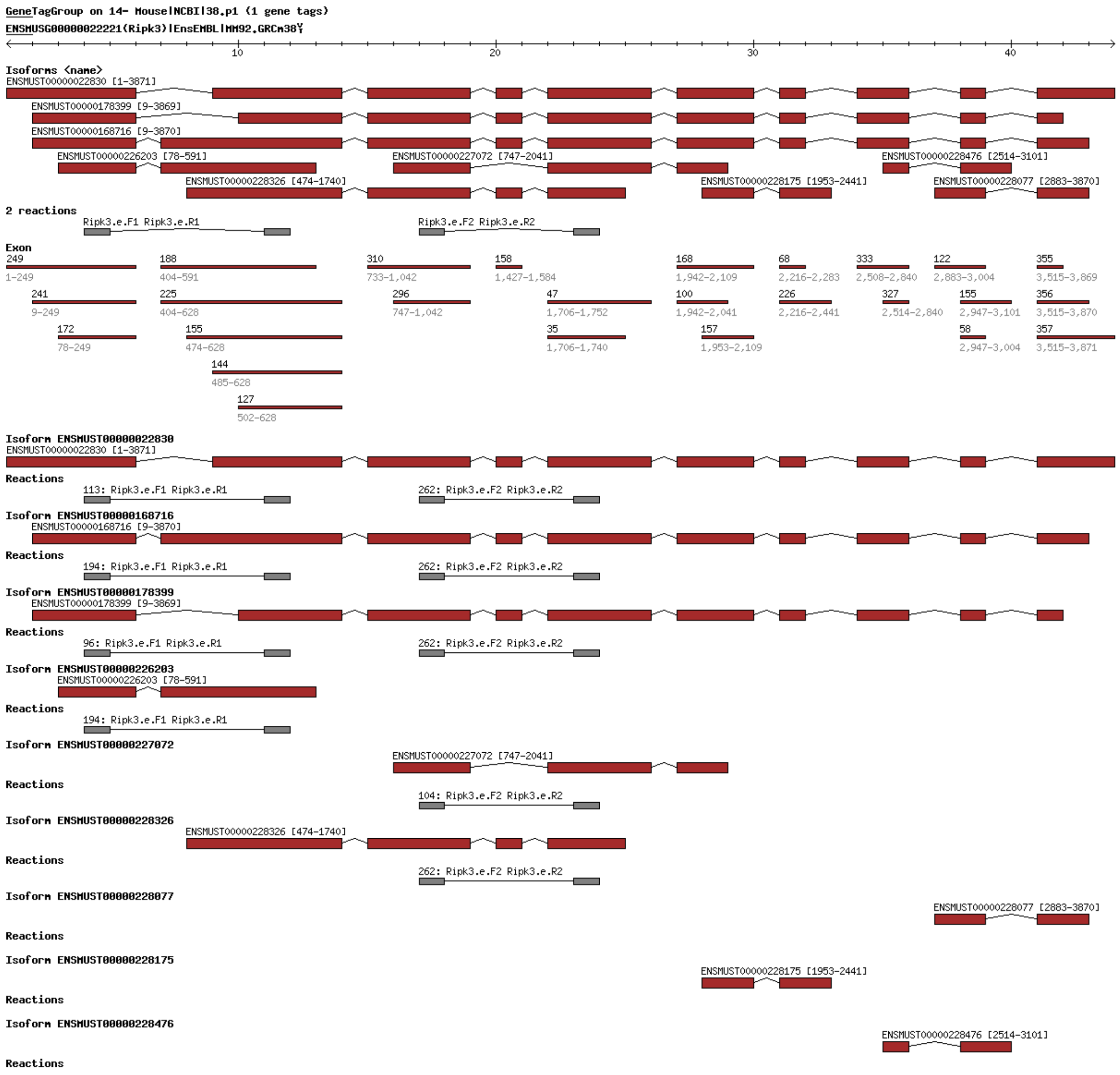

Figure S10 (cont'd)

Figure S11. Uncropped western blots from this study.

Figure 3A

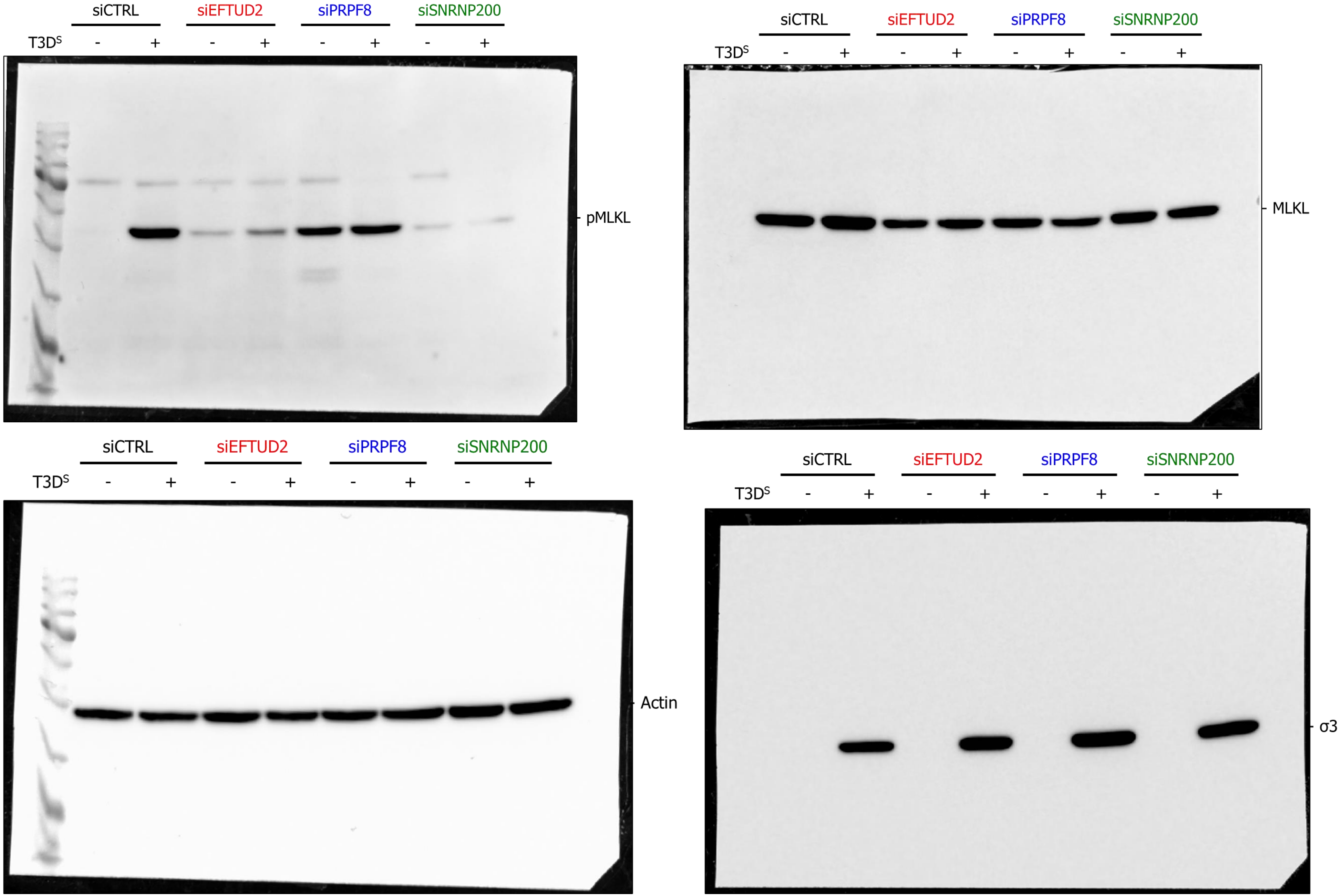

Figure S11

Figure 3D

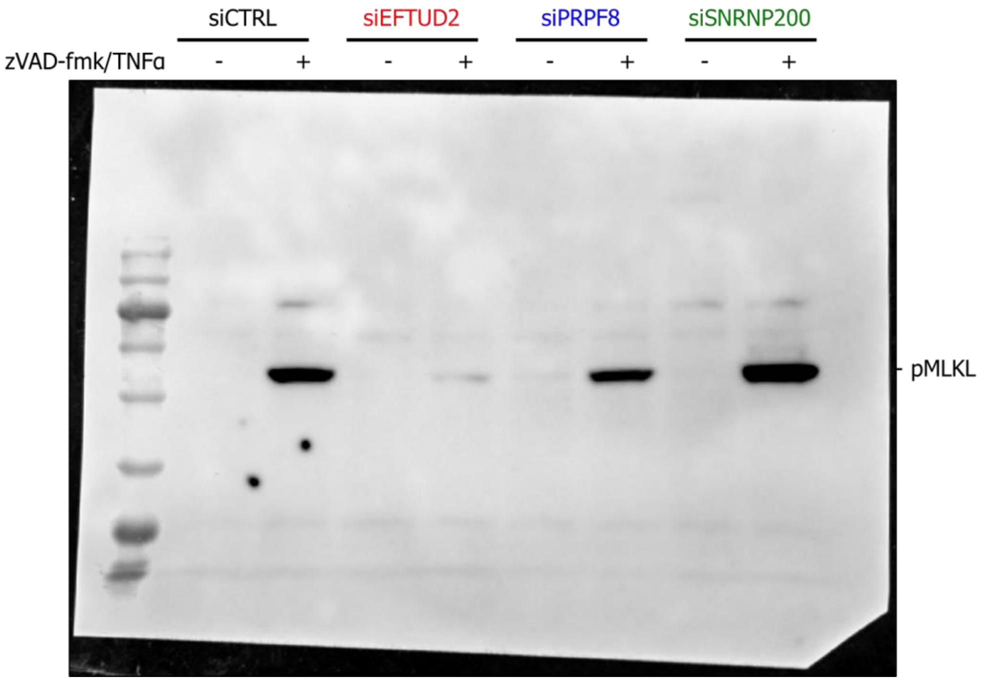

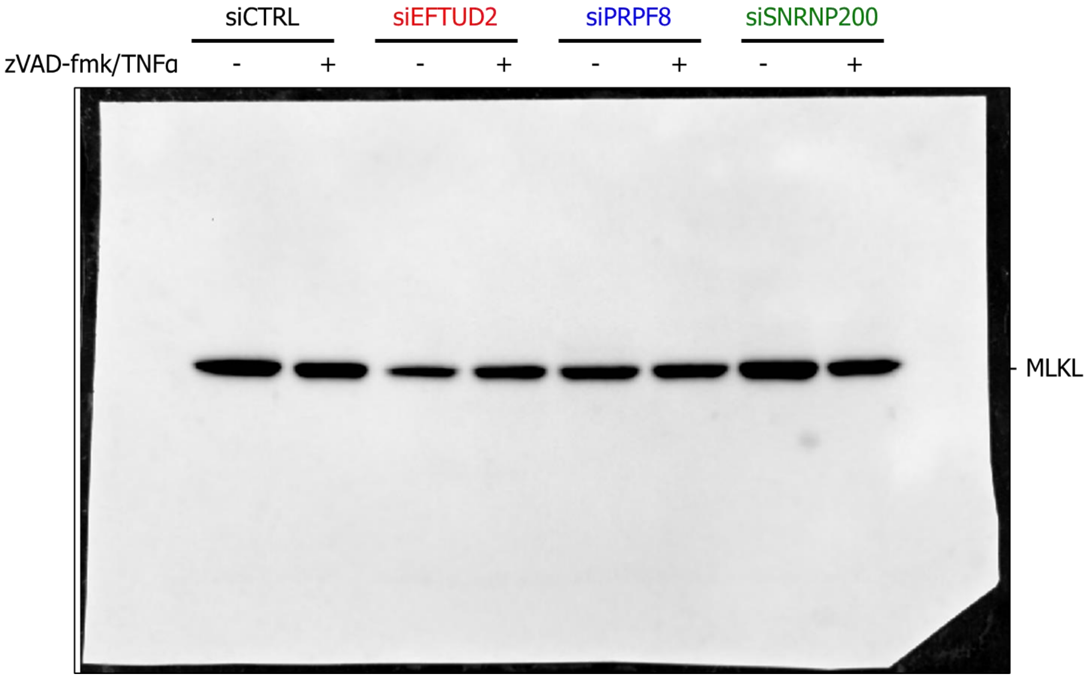

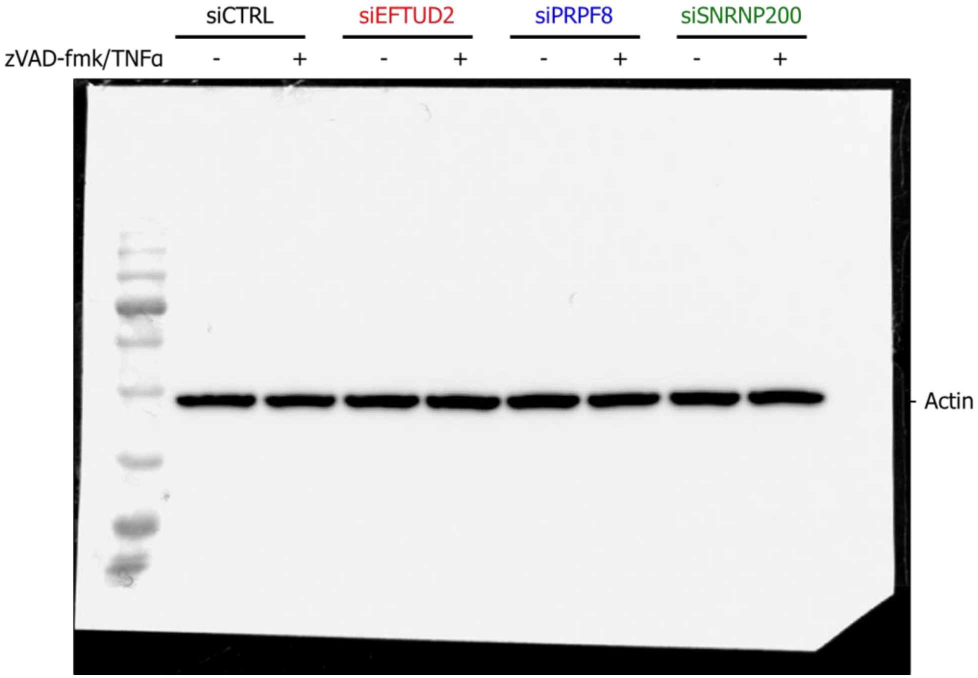

Figure S11 (cont'd)

Figure 4B

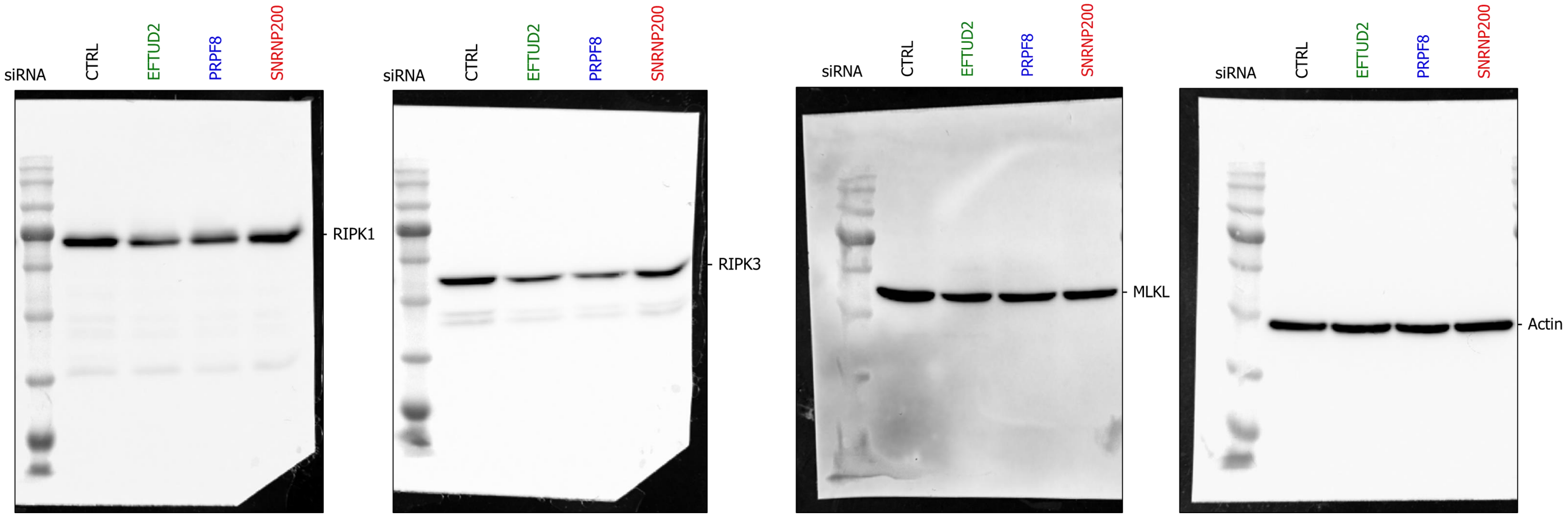

Figure S11 (cont’d)
